# Comprehensive analysis of intramolecular G-quadruplex structures: furthering the understanding of their formalism

**DOI:** 10.1093/nar/gkae182

**Published:** 2024-03-21

**Authors:** Marc Farag, Liliane Mouawad

**Affiliations:** Chemistry and Modeling for the Biology of Cancer, CNRS UMR9187, INSERM U1196, Institut Curie, PSL Research University, Université Paris-Saclay, CS 90030, 91401 ORSAYCedex, France; Chemistry and Modeling for the Biology of Cancer, CNRS UMR9187, INSERM U1196, Institut Curie, PSL Research University, Université Paris-Saclay, CS 90030, 91401 ORSAYCedex, France

## Abstract

G-quadruplexes (G4) are helical structures found in guanine-rich DNA or RNA sequences. Generally, their formalism is based on a few dozen structures, which can produce some inconsistencies or incompleteness. Using the website ASC-G4, we analyzed the structures of 333 intramolecular G4s, of all types, which allowed us to clarify some key concepts and present new information. To each of the eight distinguishable topologies corresponds a groove-width signature and a predominant glycosidic configuration (gc) pattern governed by the directions of the strands. The relative orientations of the stacking guanines within the strands, which we quantified and related to their vertical gc successions, determine the twist and tilt of the helices. The latter impact the minimum groove widths, which represent the space available for lateral ligand binding. The G4 four helices have similar twists, even when these twists are irregular, meaning that they have various angles along the strands. Despite its importance, the vertical gc succession has no strict one-to-one relationship with the topology, which explains the discrepancy between some topologies and their corresponding circular dichroism spectra. This study allowed us to introduce the new concept of platypus G4s, which are structures with properties corresponding to several topologies.

## Graphical Abstract

**Graphical Abstract:**
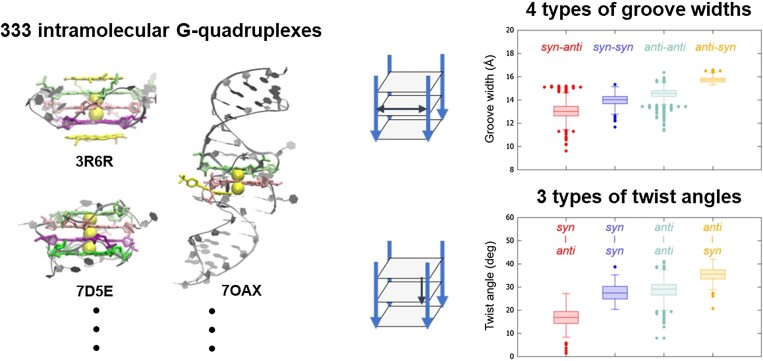

## Introduction

**Figure 1.**
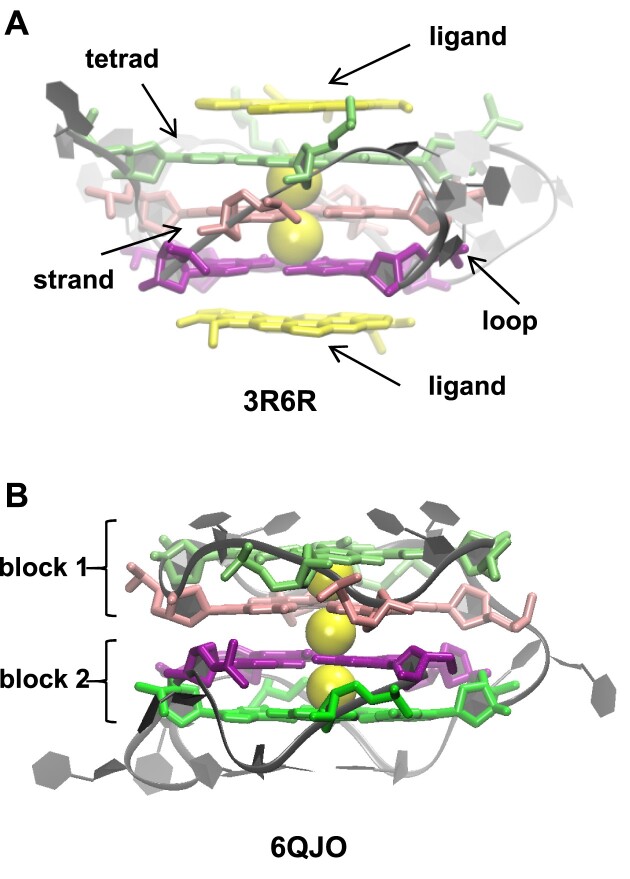
Examples of G4 structures. (**A**) A canonical G4 (PDB ID: 3R6R) and (**B**) a two-block G4 (PDB ID: 6QJO). The first tetrad is in light green, the second in pink, the third in purple, and the fourth in bright green. The rest of the G4 is gray. The K^+^ ions between the tetrads are represented as yellow spheres. In (A), the ligands on the top and at the bottom of the tetrads are yellow sticks.

**Figure 2.**
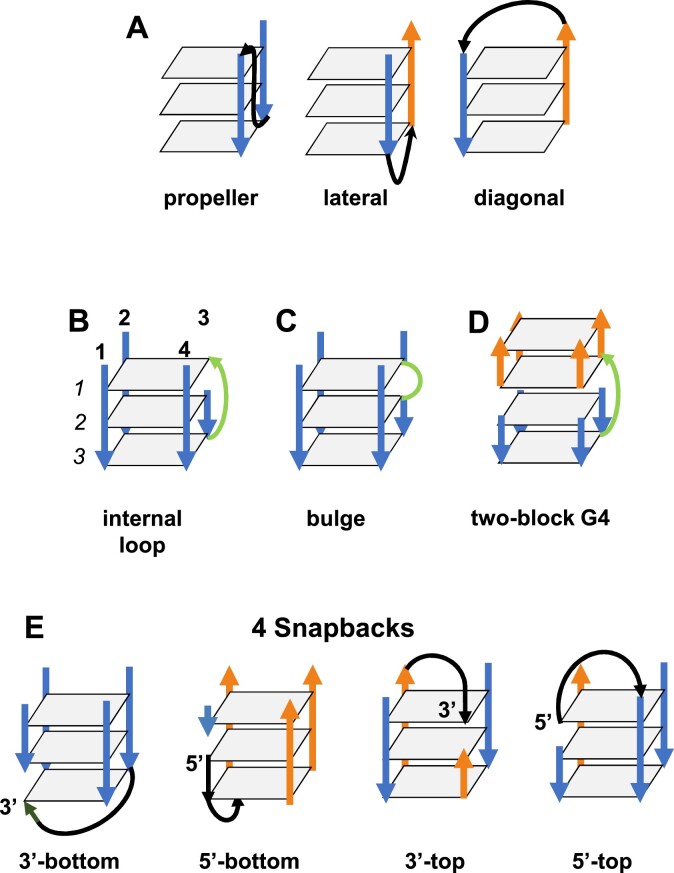
Schematic representation of the intramolecular G4 loops and discontinuities. (**A**) The three types of loops: propeller, lateral, and diagonal. (B–E) The discontinuities consist of the presence of an internal loop (**B**) or a bulge (**C**). They also exist between the two blocks of a two-block structure (**D**), or in the presence of a snapback (**E**). (A–E) The tetrads are drawn as light gray planes, the strands as arrows, blue for down and orange for up. (A) The loops are in black. For clarity, in (A) the unnecessary strands are omitted as well as the common loops in (B–E). (B–D) The internal loop, the bulge, and the linker between the two blocks are in green. (B) The numbers in bold on the top of the stem are those of the strands and the slanted numbers to the left are those of the tetrads. (**E**) There are four different types of snapbacks. The 5′ and 3′ extremities of the stem that snap back are indicated and the snapback loops are in black.

Intramolecular G-quadruplexes (G4s) are noncanonical helical structures made of one nucleic acid chain. They are present in telomeric regions, in various proto-oncogene promoters, such as *MYC* (1–12), *c-KIT* (13–16), *BCL2* (17) or *VEGF* (18), in RNA (19,20), or RNA aptamers (21–24), etc. In G4 (Figure 1), four guanines are related by Hoogsteen pairing interactions to form a tetrad, and several tetrads stack on each other to form the G4 stem. A monovalent cation, such as K^+^ or Na^+^, is coordinated to two successive tetrads, to stabilize the G4. On the edges of the stem, the successive stacking guanines form four strands, which delimit four grooves. The strands are connected by three types of loops: (i) propeller, connecting two guanosines from adjacent strands and different tetrads, (ii) lateral, connecting two guanosines from adjacent strands and the same tetrad and (iii) diagonal, connecting two guanosines from opposite strands and the same tetrad (Figure 2A). There are also two other scarce types of loops, the V-shaped and what we call here the internal loop. The V-shaped loop is a propeller loop made of 0 nucleotides (nts), i.e. it consists of only a phosphate group, whereas, the internal loop connects two non-successive tetrads within the same strand (Figure 2B). If the sequence of the G4, on its 5′ and 3′ ends, is longer than needed to constitute the stem, the additional nts are named flanking nucleotides (FN).

In the intramolecular G4 structures, a strand can present some discontinuities, meaning that the guanine identification along the strand is not continuous. A discontinuity may be due to the presence of an internal loop, a bulge, a snapback, or to the separation between the blocks in the two-block structures (Figure 2B–E). A bulge consists of the presence of supplementary nts in the strand, which are not part of the stem (Figure 2C). A two-block structure, also called G4-helix, is a G4 with discontinuities in at least three of its strands, located between the same two tetrads (Figures 1B and 2D). Whereas the internal loops are very scarce in a one-block structure, they are common in the two-block structures, where they are called linkers since they link the two separate blocks (Figure 2D). A snapback is when either the 3′ or the 5′ extremity of the stem snaps back to insert in either the first (top) or the last (bottom) tetrad (Figure 2E). Here, the stem is always viewed from top to bottom. This definition of the snapbacks results in four distinct types: 3′-bottom, 5′-bottom, 3′-top, and 5′-top.

**Figure 3.**
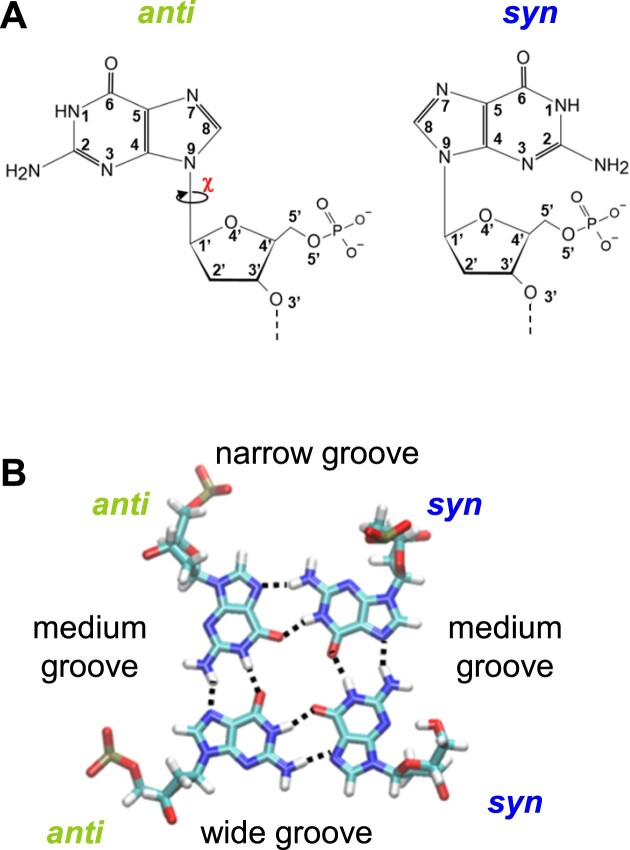
Glycosidic configurations and groove widths. (**A**) The 2D structures of *anti-G* and *syn-G*. (**B**) The 3D structure of a tetrad, made of two *anti*-Gs and two *syn*-Gs, showing the size of the corresponding grooves. The Hoogsteen H-bonds are depicted as black dashed lines. The atoms’ color code is the following: C (cyan), N (blue), O (red), P (tan), and H (white).

In the stem of the G4 structures, the glycosidic configurations (gcs), *syn* or *anti*, of the guanosines that form the Hoogsteen base-pairs (Hbps), i.e. two guanosines of the same tetrad from two adjacent strands, were described to determine the width of the groove between the two strands (25–27). The *syn–anti* and *anti–syn* Hbps result in either a narrow or a wide groove, while the *syn–syn* and *anti–anti* Hbps result in a medium groove (Figure 3). Some punctual groove width measurements based on a few structures (28,29), and a general definition (30), all using the phosphorus atoms, were published in the literature. However, the values in the latter definition are far from those in the former measurements. They are also incoherent with the histogram of the P-P distances, based on 207 G4s, that we published recently (31), hence the necessity of a systematic quantification of the groove widths. This is also important for the search for ligands targeting the grooves. Most ligands that stabilize the G4 structures are positioned on the top and/or at the bottom of the G4 tetrads (Figure 1A). However, recently, the research has become more oriented toward lateral ligands, i.e. ligands positioned in the grooves, which may allow gaining selectivity (32). Therefore, to obtain ligand specificity by targeting either wide, narrow, or medium grooves, it is necessary to calculate the groove width.

G4 structures have a limited number of topologies. Usually, five topologies are described, based on the directions of the strands: parallel, antiparallel (chair and basket), and hybrid (forms 1 and 2). They are described as being related to the gcs of the G-tetrads (30) and the groove widths (25). However, these relationships are either incomplete, as in the former, where, for example, the gc progression *anti.anti.syn.syn*, is omitted for the antiparallel-basket topology; or they contain some ambiguities, as in the latter, where, for example, the same groove width progression (mwmn) is assigned to both a hybrid and an antiparallel topology. Actually, ambiguities are observed in the five topologies themselves, especially in the hybrid forms, which are mostly given as (3 + 1) scaffold. These ambiguities are solely due to the interchangeability of the two strands adjacent to the first one. We have recently developed a website to calculate the Advanced Structural Characteristics of G4 (ASC-G4, https://institut-curie.vksolutions.io/asc-g4, (31)), in which these two strands are made distinct by always numbering them in the same direction (Supplementary Figure S1 in the Supplementary Data), following the Hoogsteen pairing and independently of the chain progression. But also, the stem is always viewed from top to bottom, and therefore, the first strand is down. These two considerations removed the ambiguities and resulted in eight perfectly distinguishable topologies depending on the direction of the strands, down (d) and up (u): parallel (dddd), antiparallel-chair (dudu), antiparallel-basket (duud), antiparallel-basket2 (dduu), hybrid1 (ddud), hybrid2 (dddu), hybrid3 (dudd) and hybrid4 (duuu). In the two-block structures (Figures 1B and 2D), each block has its own topology, among the eight. Other classification methods were proposed based either on the loops (25,33), on the relative orientation between every two stacking tetrads (30), or on arc-graphs (34). These methods are far more complex and less intuitive than the topology. Despite that, the method based on the loops is rather spread in the literature, although it is limited in scope since it only concerns canonical G4s. The canonical G4 is defined by the authors as ‘a single chain containing at least four tracts of two or more guanines that form a stem’ (33). However, there is another definition of the canonical G4, or more precisely of the non-canonical G4, which is based on its 3D structure: ‘The discontinuity of one or more G-columns (strands) in a G4 structure would make it to be considered as a non-canonical G4’ (30). Therefore, a canonical G4 has only three loops. The second definition, based on the 3D structure, is more appropriate than the first one, based on the sequence. For example, the G4 structure, PDB ID: 2MB2 (35), which has only one G-tract, d(T_2_G_15_T), would be qualified in the first definition as non-canonical despite the absence of discontinuities and the presence of only three loops. This is why, here, we adopt the second definition. In the two-block structures, which are not canonical, there can be up to nine loops. When only the three loops are considered, there are 26 hypothetical possibilities of their types and progressions, but, using a fragment-based modeling, it was shown by Fogolari *et al.* (36) that only 14 of these possibilities are physically accessible. Dvorkin *et al.* (33) combined these possibilities with vertical gc successions along the strands to propose ‘all’ possible 2- to 4-tetrad canonical G4s. This resulted in 31 G4s. However, this list is not exhaustive since there are some missing canonical G4s, like 4-tetrad parallel structures and 3-tetrad not all-*anti*-G parallel structures. Despite that, the gc successions along the strands are interesting since the circular dichroism (CD) spectra depend on them.

CD spectroscopy is widely used to determine the topology of the G4; it can only discriminate between parallel, antiparallel, and hybrid forms. The CD spectra depend on the gcs of the stacking guanosines along the strands (37,38), but no strict relationship was observed between the topology and this stacking. Indeed, Dvorkin *et al.* (33) proposed three types of gc stacking: (i) all the same gcs, (ii) two identical gcs preceded or followed by a different one and (iii) alternating gcs. In their classification, among the 31 resulting G4s, some hybrid and antiparallel topologies have the same gc stacking types (for example, in Figure 5 of reference (33), the 3-tetrad structures in line 2 of groups I (antiparallel), III, IV, V and VII (hybrid), have all the gc successions of type 2). Therefore, if the same gc successions can exist for different topologies (in this example, antiparallel and hybrid), they should yield similar CD spectra. There are also many unanswered questions about G4 structures: in which order should the gc succession along the strands be read? Does this order make any difference? G4 is a twisted helix, or more exactly four twisted helices, so, are all these helices twisted in the same way? Are all the twist angles almost equal along one helix? G4 has also tilted strands, so what are the origins of these twists and tilts? And what are their consequences?

To address all these problematics, and give answers to these questions we applied ASC-G4 to 333 publicly available G4 structures. The analyses of the results showed the importance of the unambiguous topology distinction because of the presence of a specific configuration pattern for each topology and an almost one-to-one relationship between the topology and the groove widths. It also allowed us to propose quantitative ranges for the narrow, medium, and wide grooves. In addition to that, we quantified the angles between the successive stacking guanines in the strands and related these angles to the vertical succession of gcs. This allowed us to demonstrate that the direction of reading this succession is important although it is currently not correct in the literature. Moreover, we found a correlation between these angles and the twist and tilt angles, which in turn impact the real available space in the grooves for ligand binding. All this is presented in the Results and Discussion section.

## Materials and methods

### Set of G4 structures

The list of the resolved G4 structures was downloaded from the ONQUADRO website (https://onquadro.cs.put.poznan.pl/) (39) at about the end of October 2023. It consisted of 291 intramolecular structures (named unimolecular in the website) and 154 intermolecular G4s (96 bimolecular and 58 tetramolecular). Only the intramolecular structures were kept for this study. To this list, we added 55 missing intramolecular structures that were found on the website of DSSR-G4DB (http://g4.x3dna.org) (40). From the merged list, 345 structures were downloaded from the Protein Data Bank (PDB) (http://www.rscb.org/pdb/) (41) because one structure had no available coordinates in the PDB format (7ZJ5 (42)). The downloaded structures were submitted to ASC-G4 (https://institut-curie.vksolutions.io/asc-g4) (31). Twenty did not work in the program. For eight of them, this was due to technical problems in the PDB files that were easily overcome. However, the other 12 structures had to be discarded from the set for several reasons: four structures were not real G4s, with only one tetrad (4I7Y (43), 6E82 (22), and 7ZQS (44)) or no guanine tetrads at all (6SX3 (45)); three structures were not intramolecular G4s (2WCN (46), 7ZUR (47) and 8AAD (47)); two structures were a mix of L-RNA/L-DNA (4WB2 (48) and 4WB3 (48)); and finally, three structures (7DJU (49), 7DJV (49) and 7DJW (49)), that were published in 2022, were discarded because their corresponding article was retracted in 2023 (49). This left 333 intramolecular G4 structures that were analyzed in this study. Their quality was acceptable or good considering the wwPDB validation criteria, except 2MCC (50). These structures were derived from various origins: telomers, gene promoters (*c-myc*, *KRAS*, *c-Kit*, *hTERT*, *RET*, *HIV*, etc), non-coding DNA, RNA, DNA- and RNA-aptamers, and other synthetic chains. Their sequence lengths ranged between 12 and 374 nts. Some of them were redundant, but their structures were kept in the set to investigate their ability to adopt different topologies depending on experimental conditions, like pH (51), the nature of the coordinated cation (52), and the G4 concentration (53–55).

In the NMR structures, only the first modeled frame was considered, whereas in the X-ray structures, all the G4 chains in the PDB file were analyzed, since they coexist in the crystal. Even in the presence of several chains, each PDB file is considered as one structure. Therefore, each structure is named by its PDB ID.

### ASC-G4 software

The ASC-G4 software was used locally and called automatically within a Linux loop, which allowed handling the 333 G4s in one click and less than twenty minutes. The calculated structural characteristics are the topology, the gcs of stem guanosines, the groove widths, the minimum groove widths, the helical twist angles, the tilt angles, the type, direction, and length of the loops, the directions of the strands, and the presence of bulges and snapbacks, in addition to the main chain and the sugar torsion angles. In this software, the gc is *syn* when the torsion angle ${\mathrm{\chi }} \in ] {0^\circ ,140^\circ } ]$ and *anti* when ${\mathrm{\chi }} \in ] {152^\circ ,300^\circ } ]$. This mathematical notation is equivalent to ${\mathrm{0^\circ \,<\, \chi }} \le 140^\circ$ for the former. Between these two intervals, there are two indeterminacy regions ${\mathrm{\chi }} \in ] {140^\circ ,152^\circ } ]$ and ${\mathrm{\chi }} \in ] {300^\circ ,360^\circ } ]$ where the configuration is determined based on either the distance between atoms H1’ and H8 or between atoms N3 and O5’. For more details, see (31). Because of the use of these distances to determine the glycosidic configuration of some stem guanosines, the usual GBA (glycosidic bond angle) abbreviation is not adopted in this work to designate the glycosidic configuration (gc).

Two structures have all their stem-guanosines with C1’ atom out of the base plane, 2MCC (50) and 6SUU (56), resulting in some erroneous results. 2MCC has also most of its stem-guanosines (8 over 12) with undetermined gcs and big standard deviations (SD) for almost all its structural characteristics. In addition, it is the only one-block structure with two handednesses, left-handed and right-handed twists, which seems unrealistic. Therefore, although the calculations were performed on all the structures, 6SUU was only removed from the groove width signature, whereas 2MCC was only kept for the comparison between free and liganded G4s.

For the extraction and analyses of the results, several homemade programs were used.

### Images, plots, and statistics

All structural images were drawn using the Visual Molecular Dynamics (VMD) software (57), and the plots, using Gnuplot (http://www.sourceforge.net/projects/gnuplot/) on a Windows system. The statistical tests were performed with R (http://www.R-project.org).

## Results and discussion

### Set of the studied G4 structures and some particularities

ASC-G4 was applied to the 333 intramolecular G4 structures available to date, of which 200 were resolved by NMR, 131 by X-ray crystallography, and 2 by electron microscopy; 283 G4s are DNA, 49 RNA and one is a hybrid DNA–RNA (PDB ID: 6FFR) (58); 305 structures consisted of one block and 28 of two blocks; 24 of the one-block G4s and 8 of the two-block G4s form stacking-stem dimers. The list and some of the characteristics (topology, handedness, dimerization, and snapbacks) of the one-block structures are given in Supplementary Table S1, and the two-block structures in Supplementary Table S2, in the Supplementary Data.

The large majority of the one-block structures (299/305) are right-handed helices, 5 are left-handed helices (6FQ2 (59), 7OA3 (60), 7OAV (60), 7OAW (60) and 7OAX (60)), also called Z-G4s, and one is a hybrid right-handed/left-handed helix (2MCC (50)). Of note that 2MCC, with its two handednesses, is an unusual, probably erroneous structure, showing several incoherencies, large error bars, and not obeying any of the rules found below; it is therefore excluded from the analyses, except for the comparison between the free and liganded G4s. Among the one-block G4s, there are eleven structures with some particularities. Two structures (2IDN (61) and 3QLP (62)) have a 5′–5′ inversion of polarity site, meaning that the nucleic acid chain starts with a 3′ extremity and, after a number of nts (here three), the chain is reversed with a 5′–5′ link to continue normally until the end. Seven structures are interlaced dimers (1JJP (63), 1Y8D (64), 7X7G (65), 7XDH (65), 7XH9 (65), 7XHD (65) and 7XIE (65)), meaning that, in each monomer, the first tetrad is made of three guanosines from this monomer plus one guanosine from the other monomer, to complete the tetrad. Moreover, there are two monomers with an additional guanosine that is not part of the nucleotide chain, but which inserts in the first tetrad (7MSV (66)) or the last tetrad (6K3X (67)) to complete it. These additional guanosines are not well qualified by ASC-G4 although they are recognized as part of the stem. Finally, one structure has a ligand that is intercalated between tetrads 1 and 2 (7Z9L (68)), instead of being above or below the G4 stem.

Considering the two-block structures, the presence of hybrid right-handed/left-handed helices is not scarce (8 structures over 28), as well as the left-handed helices (7/28). The remaining two-block structures (13/28) are right-handed helices.

### Comparison between G4-RNA and G4-DNA structures

Usually, in the literature, G4-RNAs are described to adopt almost exclusively a parallel fold (58,69) because rG is prone to adopt an *anti* configuration (58,69–72). However, in the subset of the one-block structures (which consists of 33 G4-RNAs, 271 G4-DNAs, and 1 hybrid G4-DNA-RNA), 15 are parallel G4-RNAs and 123 are parallel G4-DNAs. This represents the same proportion of parallel structures, 45.4%, for both G4-RNA and G4-DNA. Therefore, contrary to the common belief, G4-RNA does not seem more prone to adopt a parallel topology than the G4-DNA. However, whereas 90% of the parallel G4-DNAs consist of three tetrads and 4% of four tetrads, only 40% of the parallel G4-RNAs are made of three tetrads and none of four tetrads. Whatever the topology, the large majority of the one-block G4-RNAs (82%) consists of only 2 tetrads, despite the presence in some sequences of four G-tracts made of three consecutive guanines, like in the two 69 nt-long structures, 5OB3 (73) and 7L0Z (74). By comparison, only 30% of the one-block G4-DNAs consist of 2 tetrads. Therefore, the one-block G4-RNA seems to form more easily a 2-tetrad structure than the G4-DNA, despite the length of the chains. Indeed, in the resolved structures, the one-block G4-RNA chains can be much longer than the one-block G4-DNA chains. 70% of the G4-RNA chains are longer than 30 nts, with a maximum of 374 nts, whereas only 11% of the one-block G4-DNA chains have more than 30 nts with a maximum of 53 nts.

The case of the shortest chains, consisting of only 12 nts, is interesting because there are two structures, in the absence of ligands, with the same sequence (GGA)_4_: one is G4-DNA (1MYQ (75)) and one is G4-RNA (2RQJ (21)). They are both a dimer of parallel structures, with no significant difference between them. This is the only example of G4-DNA and G4-RNA with identical sequences and in the absence of a ligand or a protein.

In the resolved structures to date, apart from the parallel topology, the remaining 18 one-block G4-RNAs are distributed as follows: 9 structures are antiparallel-basket2 (4KZD (76), 4KZE (76), 4Q9Q (76), 4Q9R (76), 5OB3 (73), 6B14 (77), 6B3K (77), 7L0Z (74), 7ZJ4 (42)), 5 are hybrid2 (6E8S (78), 6E8T (78), 6E8U (78), 6PQ7 (79), 6UP0 (74)) and 4 are left-handed hybrid3 (7OA3, 7OAV, 7OAW, 7OAX (60)). The topologies antiparallel-chair, antiparallel-basket, hybrid1, hybrid4, and right-handed hybrid3 are still missing in the G4-RNAs. Conversely, there are two topologies that were only observed in the G4-RNAs. They concern the two-block structures.

All the two-block G4-DNA structures are made of four tetrads with topologies of the form parallel/parallel (11 structures) or parallel/hybrid2 (1 structure). They are all monomeric. In comparison, only one two-block G4-RNA structure is a monomer of the form parallel/parallel. All the other two-block G4-RNAs have no equivalent in the G4-DNAs; they consist of three tetrads with either the topology −/parallel (8 structures) or parallel/− (7 structures), where ‘−’ indicates that the block is made of only one tetrad. Four structures of each of these two topologies are dimers (see Supplementary Table S2 in the Supplementary Data).

### Four types of groove widths were found depending on the glycosidic configuration

It is generally admitted that the groove width depends on the gc of the guanosines that delimit the groove (Figure 3B) (25,27,33). This leads to the classification of medium grooves, for *anti-anti* or *syn-syn* Hbps, and narrow or wide grooves, both for *syn-anti* and *anti-syn* Hbps within the tetrad. To calculate the groove width, ASC-G4 gives the possibility to use either C5’, C3’, or P atoms. C5’ was found to be the most appropriate for the calculation of the groove width because of the presence of three peaks in the distribution of the C5’–C5’ distances (see Figure 9 in reference (31)) and the relatively small variability of these distances in each groove, with an average standard deviation < SD_C5’−C5’_> = 0.55 Å. Therefore, the distances C5’–C5’ were calculated for all tetrads in 326 structures, from which 2MCC (50) and the six platypus structures were excluded to avoid artificial inconsistencies. We call platypus structures the G4s that have heterogeneous characteristics from various topologies. For details, see below the subsection that is dedicated to the subject. The C5’–C5’ distances are all in the range 8.4 to 18.9 Å. When the C5’–C5’ distance is calculated for only one tetrad, it normally cannot be considered as the groove width, which should be the average of these distances over all the tetrads that constitute the groove. However, since the C5’–C5’ distances present a moderate variation within each groove, we consider, just in this subsection and the next one, that they may be representative of the groove width.

**Figure 4.**
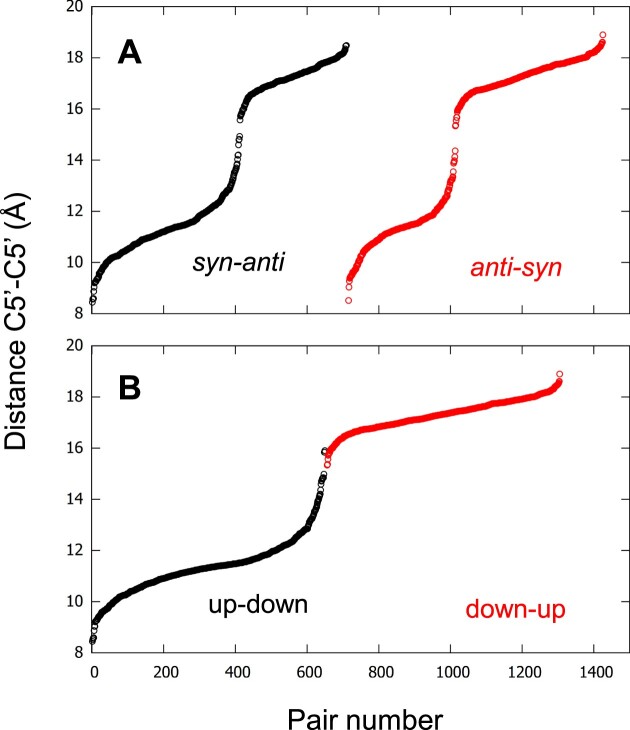
The C5’–C5’ distances, sorted according to their ascending order. The distances are calculated for all the Hoogsteen base pairs (Hbps) with the *syn-anti* configurations (black dots in (**A**)) and the *anti-syn* configurations (red dots in (**A**)), and also for all Hbps in the up-down strands (black dots in (**B**)) and the down-up strands (red dots in (**B**)). The platypus structures and 2MCC are excluded. The pair number is a sequential number attributed to each pair according to its rank in the sort. The pair numbering of the red dots starts where the pair numbering of the black dots ends.

We distributed the C5’–C5’ distances according to the guanosine configurations of the Hbps, and first focused on the *syn-anti* and *anti–syn* pairs. Their distances were separately sorted according to their ascending order, and then sequentially numbered (Figure 4A).

**Figure 5.**
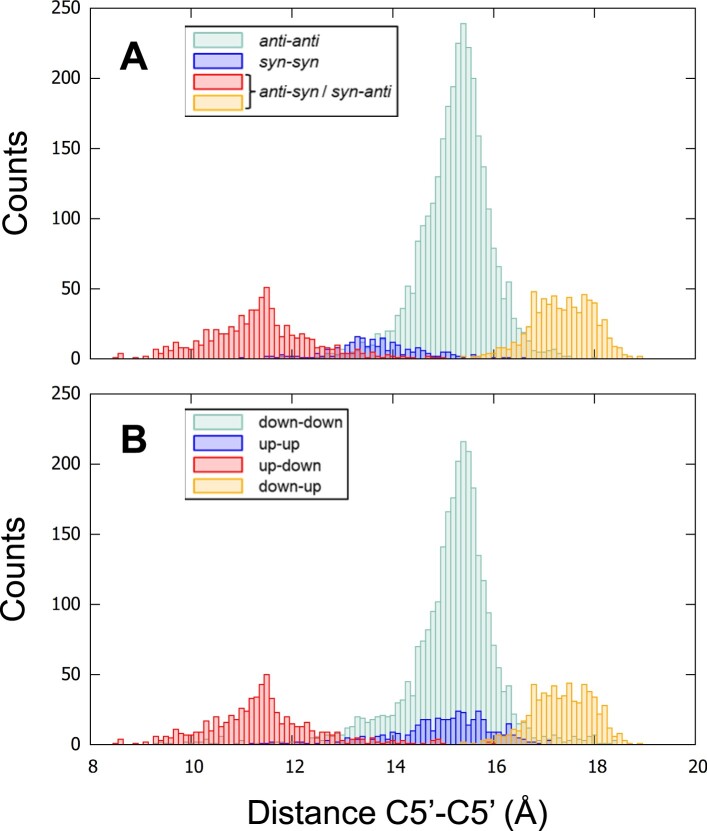
Histograms of the C5’–C5’ distances according to the gcs of Hbps (**A**) and to the direction of the strands (**B**). In (A), the small size (narrow groove) is in red, and the large size (wide groove) is in orange. Both correspond to *syn–anti* and *anti–syn* pairs with the separation distance at 15.1 Å, based on the gap observed in Figure 4A. The distribution of the *anti–anti* pairs is in pastel teal and the *syn–syn* pairs in blue. (B) The distribution up-down is in red, down-up in orange, down–down in pastel teal, and up-up in blue. The platypus structures and 2MCC are excluded from the counts. The bin size is 0.1 Å.

The distances for both types of pairs spread over the entire range of the C5’–C5’ distances, from narrow to wide passing by medium, with only a small gap in the medium range at about 15.1 Å. However, to obtain well-separated histograms of distances, the narrow and wide grooves should be delimited for the *syn-anti* and *anti-syn* pairs. For that, based on this gap, the limit was arbitrarily placed at 15.1 Å. The histograms of the C5’–C5’ distances could therefore be plotted for the four possibilities: *syn-anti*, *anti-syn* pairs, where the two types were separated in narrow (below 15.1 Å) and wide grooves (over 15.1 Å), the *syn-syn*, and *anti-anti* pairs (Figure 5A). Surprisingly, these histograms showed four distinct peaks instead of the expected three peaks that would correspond to the three types of grooves. In addition to the narrow and wide grooves, there were two separate medium grooves that we call medium-narrow for *syn–syn* and medium-wide for *anti–anti*. The *syn–syn* Hbps are not usually mentioned in the literature when describing the groove width, probably because of their rarity, as observed in Figure 5A. However, although the four distributions are well identified, there are large overlaps between them.

### The three types of groove widths were observed depending on the strand directions

Despite the identification of the four peaks, it was still unsatisfactory not to be able to distinguish between the narrow and wide grooves among these pairs, since the *syn–anti* and *anti–syn* Hbps can result in either of them (Figure 4A). However, if instead of grouping the C5’–C5’ distances according to the Hbp configurations, they were grouped according to the direction (up and down) of the adjacent strands that delimit the grooves, a clear distinction between the narrow and wide grooves would have been observed (Figure 4B, where only the up-down and down-up alternations were depicted). The up-down alternation corresponded to the narrow groove and the down-up to the wide groove. Therefore, the histograms based on the four types of adjacent strand directions were calculated simply, without the use of any artificial delimitation (Figure 5B). They showed the presence of only three peaks since the up-up peak was confounded with the down-down peak, both corresponding to the medium groove. There was only a small number of up-up adjacent strands compared to the others. Despite this clearer separation, important overlaps are still observed.

### Quantification of the groove widths

**Figure 6.**
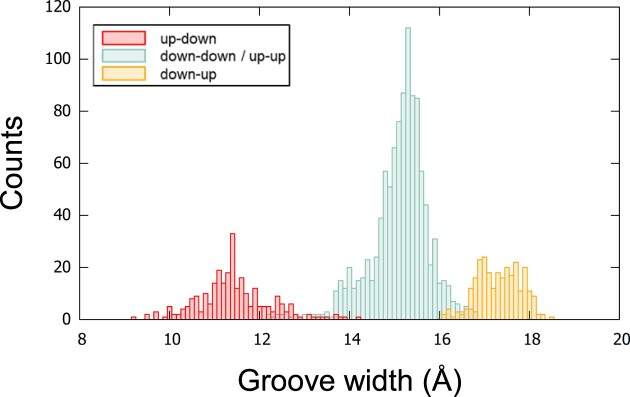
Distribution of the groove widths according to the strand directions. Up-down in red, down-down and up-up in pastel teal, and down-up in orange. The groove width is the average of the C5’–C5’ distances along each groove. The platypus structures and 2MCC are excluded from the counts. The bin size is 0.1 Å.

As stated above, there is a certain variability in the C5’–C5’ distances within the same groove. Although on average this variability is rather small, punctually it may be important. So, to quantify the groove width it is more appropriate to consider the average of the C5’–C5’ distances over the tetrads that form the groove. The distribution of these groove widths (i.e. averages) was calculated, like in the previous subsection, according to the adjacent strand directions (Figure 6). This calculation cannot be done according to the gc of adjacent guanosines because a groove is made of a variety of gcs. This distribution showed three well-separated peaks with much fewer overlaps than the similar histograms calculated for individual C5’–C5’ distances (Figure 5B). It allowed the quantification of the groove widths as follows: the narrow groove 11.4 ± 0.8 Å, the medium groove 15.1 ± 0.7 Å, and the wide groove 17.3 ± 0.5 Å. Here, the quantification of the groove width was based on the C5’ atoms. The values are different when other atoms are used. In ASC-G4, the groove widths are also given for atoms C3’ and P, therefore, the average widths for narrow, medium, and wide grooves, based on atoms C5’, C3’ and P, are given in Supplementary Table S3 in the Supplementary Data.

### Ligands and proteins do not affect significantly the groove width if the topology is not modified

**Figure 7.**
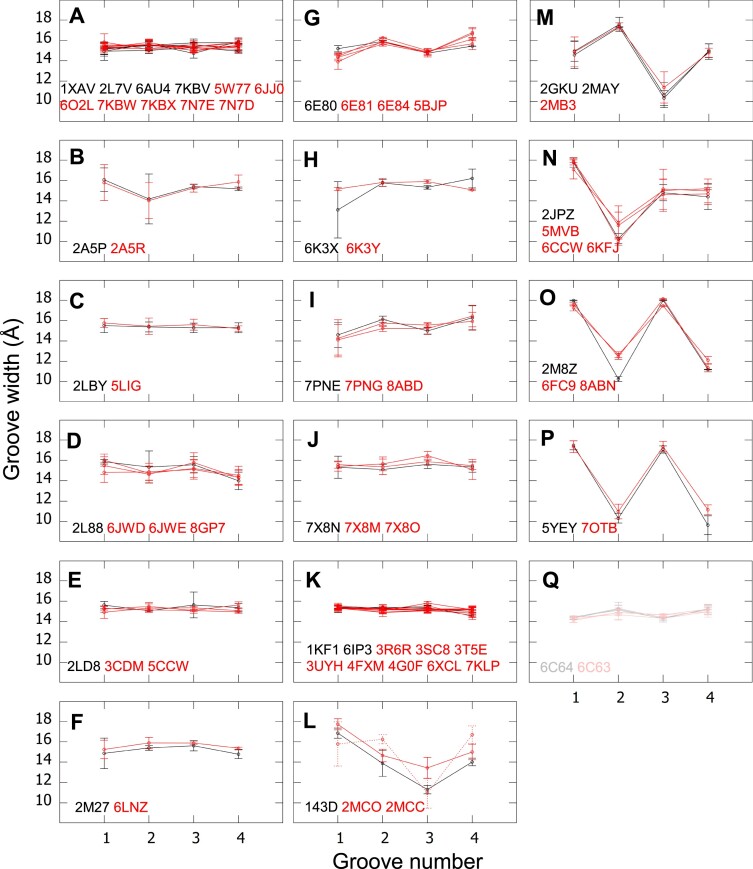
Comparison of the groove widths in the presence and absence of ligands. For each structure, the groove widths (average C5’–C5’ distances over the groove) with the SD error bars are drawn in red or pink for structures with ligands, and in black or gray, for structures without ligands (same sequence and topology). Black and red are used for the one-block G4s, and gray and pink for the two-block G4s. (L) 2MCC is drawn in dotted lines. The PDB ID is reported for each structure in the same color code. Topologies: **(A–K, Q)** parallel, **(L)** antiparallel-basket, **(M)** hybrid1 **(N)** hybrid3, and **(O, P)** antiparallel-chair. Particularities: **(G, Q)** G4-RNA and (**E**, **K–N**, **P**) telomeric G4s, among which, **(K)** and **(L)** correspond to identical sequences but different topologies.

**Figure 8.**
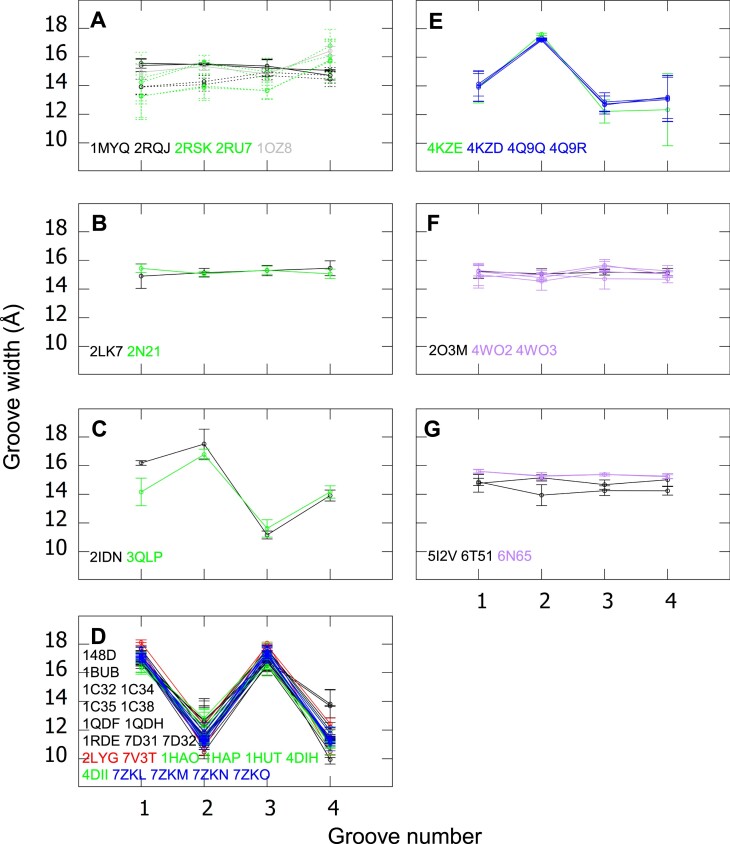
Comparison of the groove widths in the presence and absence of proteins, ligands, and dimerization. For each structure, the groove widths (average C5’–C5’ distances over the groove) with the SD error bars are drawn. In each panel, the structures have the same sequence and topology. The color code: black, one-block G4, gray, two-block G4, red, one-block G4-ligand, green, one-block G4-protein, blue, one-block G4-protein-ligand and purple, one-block G4 in dimer. The PDB IDs of the structures are reported in the same color code. Topologies: (**A, B, F, G**) parallel, (**C**) antiparallel-chair with inversion of 5′-5′ polarity site, (**D**) antiparallel-chair and **(G)** antiparallel-basket2. Particularities: (**E**) G4-RNA and (**A**) a mix of G4-DNAs (1MYQ, 1OZ8, solid lines) and G4-RNAs (2RQJ, 2RSK and 2RU7, dotted lines).

Ligands were described to sometimes induce a topology modification of the G4, by stabilizing the form with which they preferably interact (80–82). However, even in the absence of topology modification, the presence of a ligand might exert some strain and modify the G4’s groove widths. In our set, 101 G4s are bound to small molecule(s), 36 are bound to protein(s) or protein peptide(s), and 7 are bound to both, proteins and small molecules. All the remaining structures are unbound G4s. Except for one intercalated ligand (in 7Z9L (68)), all the other ligands are located on the top of the stem and/or at its bottom. To investigate the possible effect of binding ligands and proteins on the groove widths, we compared the average C5’–C5’ distances along the grooves, by grouping in each panel of Figures 7 and 8 the structures with identical sequences and topologies that existed in our set in both the presence and absence of a ligand/protein (i.e. 43 liganded G4s, 10 G4s in complex with a protein and 7 G4s in complex with both). In Figure 7, the comparison between the groove widths of liganded (red curves) and unliganded (black curves) G4s show no significant difference, whether they be one-block (panels A–K, M–P) or two-block (panel Q) structures. The only exception is in panel L, where one liganded structure presents significantly different groove widths from the two others, 2MCC (50). The curve of 2MCC (red dotted line) is even different from that of the other liganded structure, 2MCO (50) (red solid line), and it presents large error bars, reflecting its poor quality. This is one of the reasons that led us to exclude it from all the other analyses. Despite that, focusing on panels K and L (discarding 2MCC) is interesting because they concern telomeric G4s with exactly the same sequence but different topologies. In panel K, all structures (29,83–89) were resolved using X-ray crystallography, in the presence of K^+^ and polyethylene-glycol, and their topology is parallel, whereas in panel L, the structures (50,90) were resolved using NMR, in the presence of Na^+^, and their topology is antiparallel-basket. The difference in topology results in a much more important difference in the groove widths than the presence or absence of a ligand.

In Figure 8, where we investigate the effects of protein binding and dimerization, there are two panels that also compare structures with and without ligands: panel D, where all possibilities are represented, i.e. free G4s and the complexes, G4-ligand, G4-protein, and G4-protein-ligand and panel E, which compares the complexes G4-protein and G4-protein-ligand. Here too, there is no significant effect of the ligand on the groove widths. No significant difference in the groove widths was observed either upon the binding of a protein, as seen in Figure 8A–D.

The case of Figure 8A is interesting because it concerns G4-RNAs and G4-DNAs, with the same sequences. These structures are stacking-stem top-to-top dimers either free, 1MYQ (75) (G4-DNA) and 2RQJ (21) (G4-RNA), or in a complex on each side of the dimer with two identical protein peptides, 2RSK (23) and 2RU7 (24) (both are G4-RNAs and have the same peptides), and also an equivalent free two-block structure, 1OZ8 (91) (G4-DNA) with no protein or ligand, but in which the two monomers are linked by a covalent bond. One observes some differences in the groove widths, but they are of the same amplitude as the differences between the two similar G4-protein-peptide complexes, 2RSK and 2RU7 (green curves). Therefore, they cannot be imputable to the difference of nature (DNA/RNA), the presence of protein peptides, or the presence of a linker between the two monomers. They are probably due to the resolution of the structures or their thermal fluctuations.

There are only two sets of structures in which the G4 exists in two forms: a monomer and a stacking-stem dimer. As observed in Figure 8F,G, the dimerization also does not have any significant effect on the groove widths.

### A groove-width signature specific to each topology

**Figure 9.**
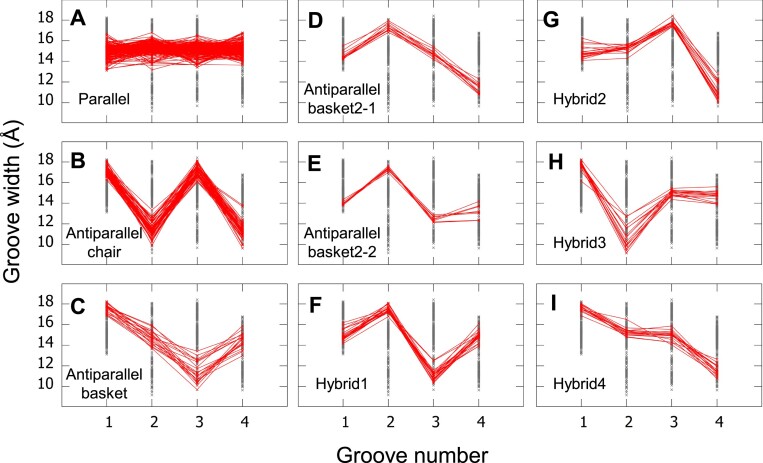
A groove-width signature for each topology. In each panel, the groove widths calculated from atoms C5’ of all the one-block and two-block structures are reported as dark gray crosses, and only for the indicated topology the corresponding crosses are connected with red lines. The parallel topology includes parallel/parallel, parallel/−, and −/parallel structures. The antiparallel-basket2-1 signature corresponds to G4-DNAs and antiparallel-basket2-2 to G4-RNAs.

Since it was shown above that the groove width i.e. the average C5’–C5’ distances, depends on the direction of the strands, which in turn defines the topology, we investigated the presence of a groove-width signature for each topology. To this purpose, for each of the eight topology types, the groove width was drawn for all the grooves of the structures as gray crosses, and for each topology, the four groove widths of the corresponding structures were related with red lines (Figure 9). In the parallel topology, we included the two-block structures parallel/parallel, −/parallel, and parallel/−. As observed, there is a unique signature of the groove width, representative of each topology, except for the antiparallel-basket2, which has two distinct signatures. These signatures are also given in Tables 1 and 2: iiii for parallel, wnwn for antiparallel-chair, wini for antiparallel-basket, iwin and iwii for antiparallel-basket2, iwni for hybrid1, iiwn for hybrid2, wnii for hybrid3, and wiin for hybrid4, (where n stands for narrow, i for intermediate size, and w for wide groove; here i was preferred to m, for medium, for clarity because the letter m is hardly distinguishable from n and w). The balance between i, n, and w in each of these groove-width signatures results in an almost constant perimeter of the stems, that is the sum of four medium grooves. The average perimeter calculated from the C5’ atoms of all structures is 59.4 ± 2.3 Å. We also calculated this perimeter based on C3’ atoms, which is 57.5 ± 1.6 Å, and on P atoms, which is 62.6 ± 4.3 Å. In the antiparallel-basket2 topology, there are two distinct signatures: iwin, which is only observed in the G4-DNAs, and iwii, which is only observed in the G4-RNAs. In the latter signature, iwii, the balance seems broken, but this is not the case since the perimeters in the corresponding structures are in the same range as the others. This is because the last two grooves have their widths between intermediate and narrow. The iwii signature is found in nine very long G4-RNA sequences of 69 nts (5OB3 (73), 7L0Z (74)), 83 nts (6B14 (77), 6B3K (77)), 84 nts (4KZD (76), 4KZE (76), 4Q9Q (76), 4Q9R (76)) and 374 nts (7ZJ4 (42)). The particularity of this signature is probably due to the presence of relatively long dsRNA structures on both sides of the G4. This seems to be corroborated by the fact that the hybrid2 G4-RNAs, with much shorter sequences (36–38 nts) and a dsRNA on only one side of the G4, do not present any particular groove-width signature; idem for the parallel G4-RNA structures (12–49 nts), whether they be one-block or two-block G4s, with only a one-side dsRNA, when it exists.

**Table 1.** The G4 topologies of the 305 one-block structures (including 2MCC) and their corresponding strand directions, gc patterns, groove-width signatures, and loop types. In the ‘loop combinations (occurrence)’ column, for each topology, the loop combinations are ordered from the highest occurrence to the lowest, the occurrence (in parentheses) being the number of structures where the combination was found. In the presence of a snapback, the quadruplex has four loops. The loop order in each combination (the last column) follows the nucleotide chain sequence, whereas the order of the strands, the gcs, and the groove widths (the other three columns) follows the Hoogsteen pairing. In Hybrid1, we added the structure with the undetermined topology, 1JJP (63), because of its gc pattern and groove-width signature

Topology	Strand direction^a^	Gc patterns in tetrads in the absence of strand discontinuities^b^	Groove-width signature^c^	Loop combinations (occurrence)^d^
**Parallel (138)**	dddd	aaaa, ssss	iiii	-p-p-p (118), -p-p-p*d*^e^ (7), -p-p-l*+p* (5), -p-p-p-*l* (4), *-p*-p-p-p (2), -p*i*^f^*-*l-p (1), *+l*-p-p-p (1)
**Antiparallel-chair (63)**	dudu	sasa, asas	wnwn	+l+l+l (54), -l-l-l (9)
**Antiparallel-basket (22)**	duud	saas, assa	wini	-ld+l (20), d+pd (2)
**Antiparallel-basket2 (15)**	dduu	ssaa, aass	iwii, iwin	-pd+p (9), +ld-l (5), +l+p+l (1)
**Hybrid1 (15+1(1JJP))**	ddud	ssas, aasa	iwni	-p-l-l (14), *+l*-p-l-l (1), -p-l-p (1JJP)
**Hybrid2 (14)**	dddu	sssa, aaas	iiwn	-pd+l (6), -p-p-l (6), +ld-p (1), -l-p-l*d* (1)
**Hybrid3 (21)**	dudd	sass, asaa	wnii	-l-l-p (17), *-l*+l+p+l (4)
**Hybrid4 (16)**	duuu	saaa, asss	wiin	*-l*+l+p+p (8), *d*+p+l+p (4), +l+p+p (2), +l+p+p+*l* (2)

^a^d stands for down and u for up.

^b^a stands for *anti*-G and s for *syn*-G.

^c^n stands for narrow, i for intermediate (or medium), and w for wide groove

^d^p stands for propeller, l for lateral, and d for diagonal.

^e^Loops in italics are due to the presence of a snapback: at the beginning of the combination they are 5′-snapbacks (5′-bottom or 5′-top), at the end, they are 3′-snapbacks (3′-bottom or 3′-top).

^f^The exceptional internal loop (*i*) from bottom to top in strand 2 (structure 2M53 (94), see Figure 2B).

**Table 2.** The G4 topologies of the 28 two-block structures and their corresponding strand directions, gc patterns, groove-width signatures, and loop types. In the ‘loop combinations (occurrence)’ column, for each topology, the loop combinations are ordered from the highest occurrence to the lowest, the occurrence (in parentheses) being the number of structures where the combination was found. The loop order in each combination (the last column) follows the nucleotide chain sequence, whereas the order of the strands, the gcs, and the groove widths (the other three columns) follows the Hoogsteen pairing

Topology^a^	Strand direction^b^	Gc patterns in tetrads^c^	Groove-width signature^d^	Loop combinations (occurrence)^e^
**Parallel / Parallel (3)**	dddd / dddd	aaaa / aaaa	iiii	-p-p-p+p+p+p+*p*^f^ (2), -p-p-p-p-p-p (1)
**Parallel / Parallel (7)**	dddd / uuuu	aaaa / aaaa	iiii	-p-p-p+p+p+p+*p* (3), +p+p+p-p-p-p-*p* (2), -p-p-p+*p*^g)^+p+p+p (1), -*p*-p-p-p+*p*^g^+p+p+p+*p* (1)
**Parallel / Parallel (2)**	uuuu / dddd	aaaa / aaaa	iiii	-p-p-p+p+p+p (2)
**Parallel / Hybrid2 (1)**	uuuu / dddu	s^h^aaa / aaas	iiii / iiwn	-*p*-p-p-p+p+p+*l* (1)
**Parallel / − (7)**	dddd / one-tetrad	aaaa / aaaa(s)^i^	iiii	-p-p-p (6), -p-p-p-p (1)
**− / Parallel (8)**	one-tetrad / uuuu	aaaa(s)^i^ / aaaa	iiii	-l+p+p+p (4), +p+p+p+p-p (4)

^a^Topology in block1 / block2. In all the Table, the slashes separate the two blocks.

^b^d stands for down and u for up.

^c^a stands for *anti*-G and s for *syn*-G.

^d^n stands for narrow, i for intermediate (or medium), and w for wide groove.

^e^p stands for propeller, l for lateral.

^f^Loops in italics are due to the presence of a snapback: at the beginning of the combination it is a 5′-snapback (5′-bottom or 5′-top) at the end, it is a 3′-snapback (3′-bottom or 3′-top).

^g^The loop in italics in the center of the combination links two strands one from each block (structure 6GZ6 (59)).

^h^s is due to the presence of a 3’-top snapback.

^i^In some structures the last gc of the one-tetrad block is s.

Eleven structures were excluded from the plots in Figure 9 because they did not fit the topology signatures: 6KVB (92), which is a two-block structure with the mixed topologies parallel/hybrid2, could not fit in either of them because the average is done on the entire groove; 1JJP (63), which is an interlaced dimer where each monomer is made of two tetrads, has an undetermined topology because one of its strands consists of one nt from each chain, and has therefore an undetermined direction; 2MCC (50) (basket), which was mentioned above, because it presents obvious errors; 6SUU (56) (parallel) because of its poor quality since all its stem guanine bases are not planar. The other seven structures (5J05 (33), basket; 6JCD (93), hybrid2; 2MFU, 7OA3 (60), 7OAV (60), 7OAW (60) and 7OAX (60), hybrid 3), unlike the first four, fit rather well the topology signatures based on C3’ atoms (Supplementary Figure S2A-C in the Supplementary Data), probably because C3’ is part of the sugar and has less latitude of movement than C5’.

The presence of strand discontinuities due to bulges and snapbacks or the separation in the two-block structures does not modify the groove-width signature, although it modifies in some cases the configurations (see below). In Figure 9, we can note the absence of points from any structures in the narrow range in the first groove and the wide range in the fourth groove. This is because the first groove is delimited by strands 1 – 2, and, in the one-block structures, by definition strand 1 is always down, and strand 2 can be either down, resulting in down-down directions and therefore, a medium groove, or up, resulting in down-up directions, and therefore, a wide groove (see Figure 6 for the histograms of the groove widths). So, the up-down directions, which would result in a narrow groove, do not exist. Considering the fourth groove, it is delimited by strands 4 – 1. This leads to either down-down directions, and therefore, a medium groove, or up-down directions, and therefore, a narrow groove, so the wide groove does not exist. In the two-block structures, the first strand in one of the blocks can be up, but in our set, all the two-block structures, except 6KVB, are parallel, with the strand directions in each block either dddd or uuuu, resulting in medium grooves. 6KVB, which was excluded from Figure 9, is parallel/hybrid2, with the strand directions uuuu/dddu. Therefore, in the first block, all grooves are delimited by directions uu, corresponding to the medium size, whereas in the second block, the grooves are delimited as follows: groove 1, dd, groove 2, dd, groove 3, du and groove 4, ud. Therefore, the average groove width along the two blocks is medium for grooves 1 and 2, it is between medium and wide for groove 3 and between narrow and medium for groove 4. Consequently, for all the 2-block structures, there is no narrow groove 1 or wide groove 4.

### A predominant gc pattern of adjacent guanines specific to each topology in regular G4s

**Figure 10.**
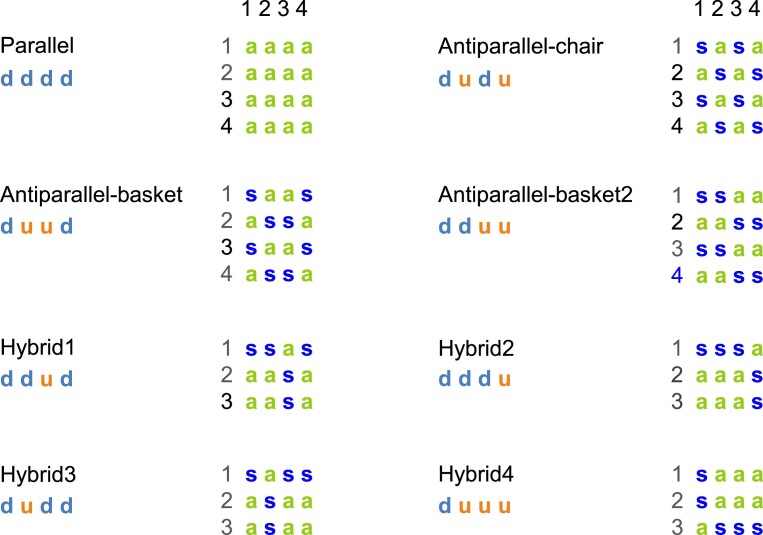
The gc pattern of the longest one-block structures for each topology in regular G4s. The glycosidic configurations of the guanosines, a, for *anti* (green) and s, for *syn* (dark blue), are preceded by the number of the tetrad (black). Under the topology, the strand directions are given: d, for down (light blue) and u, for up (orange). In the top horizontal line, the strand numbers are given. These patterns are observed in the one-block structures with the biggest number of tetrads, without discontinuities, like bulges and snapbacks, i.e. in canonical G4s.

In regular G4s (i.e. one-block right-handed G4s without 5′–5′ inversion of polarity site, 297 structures), the analysis of the gcs of all stem guanines showed the presence of a predominant specific pattern for each topology (Figure 10 and Table 1). The horizontal progression (in a tetrad) of the *syn* (s) and *anti* (a) configurations of adjacent guanines is similar to the progression of the strand directions, down (d) and up (u). In other words, along a tetrad the two adjacent guanines have different configurations (*syn-anti* or *anti-syn*) when the adjacent strands have different directions (down-up or up-down) and they have the same configuration (*syn-syn* or *anti-anti*) when the adjacent strands have the same direction (up-up or down-down). This explains why the groove widths, which are dependent on the gcs of adjacent guanines, are also correlated to the directions of adjacent strands.

### The reasons for the correlation between the strand direction and the gc pattern of a tetrad

In regular G4s, when looking at a strand in its 5′→3′ direction, the *syn*-G orients its H-bond donors to the right (over the ribose) and the *anti*-G to the left (avoiding the ribose) (Figure 3A). So, if the first guanine of a tetrad (i.e. belonging to the first strand, which is always oriented downward) is *anti*, its H-bond donors and those of all the other guanines of the same tetrad are oriented to the left to follow the Hoogsteen pairing (Supplementary Figure S1 in the Supplementary Data). Therefore, if among these guanines there is a *syn*-G, its H-bond donors should be oriented to the left as well. However, to respect the rule of its orientation to the right relative to its own strand, this strand should be reversed. Similarly, if the first guanine of a tetrad is *syn*, all the H-bond donors are oriented to the right, and an *anti*-G can only insert in the Hoogsteen pairing if it belongs to a reversed strand. The reasoning is the same starting from any guanine of the tetrad, not only the first one, as long as we look at the corresponding strand from top to bottom according to its own direction (not the direction of the stem). This explains the correlation between the progression of the strand directions and that of the gcs in the tetrads.

In regular G4s, there are some apparent exceptions where this correlation seems to be broken. This is the case when there is a discontinuity in a strand, allowing two opposite directions within this strand, like in the presence of a long bulge, or a snapback. Indeed, this is illustrated in Supplementary Figure S3, in the Supplementary Data, by the example of three parallel structures, which would normally consist of all *anti* stem-guanosines in addition to a hybrid4 structure, whose last tetrad should be of the form asss (Figure 10). Yet, the presence of a long bulge, as in 7CLS (95) (Supplementary Figure S3A), or a 3′-bottom snapback, as in 2MGN (10) (Supplementary Figure S3B), results in the existence of a *syn*-G in the bottom tetrad because the loop which bears this *syn*-G is reversed relative to the rest of the strand. Contrarily, if the 3′-bottom snapback consists of two guanosines instead of one, as in 2O3M (13) (Supplementary Figure S3C), the snapback can be made of *anti*-G instead of *syn*-G because its length gives it the possibility of adopting the regular down strand orientation. The example of the hybrid4 structure, 6RS3 (96) is more complex because the gc inversion does not take place in the strand with the discontinuity, but in its adjacent strand (Supplementary Figure S3D). In hybrid4 structures that do not present a 5′-bottom snapback in their strand 1, the gcs of the last tetrad is unambiguously either asss or saaa. However, in the presence of the 5′-bottom snapback, strand 1 consists then of nts (X − 1 − 2) from top to bottom and strand 4 of nts (X+1 − X+2 − X+3) from bottom to top. Therefore, strands 1 and 4 are connected with a 0-nt propeller loop between nts X and X+1. By the way, this loop is not V-shaped, while the V shape is observed within strand 4 itself, as for instance, between DG15 and DG16 in 6RS3 (Supplementary Figure S3D). The shortness of the 0-nt loop results in a down micro-direction at nt X+1, which is at the junction of strand 4 and tetrad 3. Consequently, its gc is sometimes on the edge between *syn* and *anti* and it is considered *syn* by ASC-G4 (which makes asss), or it is clearly *anti* (which makes assa), as in 6RS3.

Based on all these observations, the same rule of the guanine orientation as above is followed, but more precisely, the correlation is between the gc progression in the tetrads and the micro-directions of the strands instead of their global directions.

Qualifying the groove width according to the adjacent strand directions rather than the gcs of Hbps has several advantages, first, the distances of narrow and wide grooves are better delimited (Figure 4), second, the two strands delimiting a groove have only two directions (except in the two-block structures), but may have a mix of adjacent gcs due to the presence of bulges and snapbacks (Supplementary Figure S3 in the Supplementary Data). Third, the groove width can be predicted from the strand directions much more straightforwardly than from the gcs, especially in case of *syn-anti* or *anti-syn* Hbps. And finally, the groove width, based on the strand directions, explains the presence of a unique groove-width signature for each topology. However, in some cases, relying solely on gcs is necessary, like for the structure 1JJP (63), which is a 2-tetrad interlaced dimer, with an unknown topology due to its strand 3. This strand consists of one nt from each chain, which makes its direction undetermined. In this case, despite the discontinuity in strand 3, the gc pattern gives the hint about the topology. Indeed, in 1JJP, this strand is made of two *syn-*Gs (one from each chain) and the G4 has, therefore, the gc pattern of the hybrid1 topology, aasa, for both tetrads. Its groove-width signature too is that of a hybrid1 structure (Supplementary Figure S2E, in the Supplementary Data). Therefore, it was classified in Table 1 as a hybrid1 structure. By comparison, all the other interlaced dimers or monomer are 3-tetrad parallel structures, with the gc pattern aasa for the first tetrad due to the discontinuity (the s is the gc of the nt from the other chain) and aaaa for the other two tetrads; their groove-width signatures are those of parallel structures as well.

### The topology and groove widths can be deduced from only one tetrad in regular G4s and in the absence of discontinuities

Remarkably, the knowledge of the gc pattern allows us to deduce unambiguously the topology of a regular G4, just from the visual observation in the top-to-bottom direction of the stem of only one tetrad. This can be any tetrad provided it does not contain any guanine from a strand discontinuity (snapback, long bulge, internal loop, or a nt from another chain) and provided we know which is the first strand. Since the first strand is always down, with the strand numbering following the clockwise direction, and since the progression of *syn*-G and *anti*-G corresponds to the progression of the up and down strands, the topology can be deduced from the visual detection of the *syn* and *anti* guanines of the tetrad, by using Table 1. The knowledge of the topology suffices to determine the groove-width signature and therefore, the approximate values of the groove widths, without any calculations. Two examples are given in Supplementary Figure S4, in the Supplementary Data.

### Classification of G4s based on their loops

The classification method based on the loops (25,33) was described to only apply to canonical G4s, which are defined as a single nucleotide chain with at least four tracts of guanines that form a stem without discontinuities. Therefore, the canonical G4 contains only three loops. In this classification method, the loops are characterized by their type, propeller (p), lateral (l) or diagonal (d), and their progression, clockwise (+) or anticlockwise (–). The progression of the loops depends on the orientation of the G4, which must be from bottom to top (25). In ASC-G4, although the G4 is viewed from top to bottom (without incidence on any of the calculated characteristics), the loop progression is provided according to the bottom-to-top orientation, to be in concordance with previous publications. Dependency of loop progression, ‘+’ and ‘–’, on G4 orientation produces aberrant effects. The example of the canonical structure 1MYQ (75), and its equivalent two-block structure 1OZ8 (91), mentioned above in Figure 8A, is illustrative. 1MYQ, whose sequence is d[(GGA)_4_], is a canonical parallel structure, in a top-to-top dimer (meaning that tetrad 1 of both chains are in close contact), and 1OZ8, whose sequence is twice as long, d[(GGA)_8_], is a two-block structure corresponding to the covalently connected 1MYQ dimer (see Supplementary Figure S5, in the Supplementary Data). If we arbitrarily consider one chain of 1MYQ, and orient it from bottom to top, its loop classification is -p-p-p, but the other chain, which has a reversed orientation within the dimer, i.e. from top to bottom, has its loop progression +p+p+p. However, there is no objective reason why its loop progression should be opposite since this chain has the same probability to be chosen as the first one, and then its loop progression would be -p-p-p. Therefore, in ASC-G4 the two chains are disconnected and oriented from bottom to top and have the loop progression -p-p-p. But, in 1OZ8, where the chains are covalently connected, there is no way to split them and the loops are -p-p-p+p+p+p. Therefore, there is a dilemma, either one chooses to have one chain in the dimer with an incorrect loop progression, or one accepts that the dimer and the two-block structure do not have the same loop progressions, although they are similar. This example illustrates well the problem of using a classification method that is dependent on the global orientation of the G4 in space.

Nonetheless, in ASC-G4, where all G4s are handled, all loops are given, even those concerning the snapbacks (see Table 1) and the two-block structures (Table 2). Their number can be up to 9 loops for a two-block structure, where their progression may be opposite in the two blocks, depending on their relative orientation. One can distinguish between the loops that are due to snapbacks (in italics in Tables 1 and 2) and the basic loops, i.e. the usual loops that connect strands. The 14 loop combinations predicted by Fogolari *et al.* (36) were found in the 305 one-block structures. However, we found three additional basic combinations that were not predicted. They only exist in the presence of discontinuities: -p-l-p, in the 2-tetrad interlaced dimer (1JJP (63)), -l-p-l (followed by the snapback loop *d*) in a hybrid2 G4 (6JCD (93)), and +p+l+p (preceded by the snapback loop *d*) in four hybrid4 G4s (5ZEV (97), 6H1K (98), 7YS5 and 7YS7). Besides, we found two basic loop combinations that exist in two different topologies, provided the presence of a snapback in one of the two topologies: -p-p-l in hybrid2 and parallel (followed by +*p* in parallel, 2M92 (99), 2O3M (13), 3QXR (100), 4WO2 (101) and 4WO3 (101)) and +l+p+l in basket2 and hybrid3 (preceded by -*l* in hybrid3, 7OA3 (60), 7OAV (60), 7OAW (60) and 7OAX (60)).

One should be aware that the loop order in Tables 1 and 2 follows, as usual, the nucleic acid chain direction. This is not the case of the order of the strands, which follows the Hoogsteen interactions. The latter impacts the order of the strand directions, and consequently the topology, the gc patterns, and the groove-width signature. To illustrate the difference between the order of the strands and the chain direction, we will take the example of 2MBJ (102). In Table 1, this is the only antiparallel-basket2 structure, with the loops +l+p+l. By following the Hoogsteen pairing, the resulting strands have the following identifications (position of the guanosines in the nucleotide chain): strand 1 = (4 − 5 − 6), strand 2 = (22 − 23 − 24), strand 3 = (18 − 17 − 16), and strand 4 = (12 − 11 − 10). In each strand, the first number corresponds to a guanosine in tetrad 1 and the last number, in tetrad 3. From the order of the identifications we observe that the first two strands are down and the last two are up, and therefore, the groove-width signature is iwin. However, this does not mean that the first groove (width i) has a lateral loop, the second (w) a propeller loop and the third (i) a lateral loop. Actually, loop 1 (+l) connects strands 1 and 4, loop 2 (+p) connects strands 4 and 3, and loop 3 (+l) connects strands 3 and 2. Therefore, because the Hoogsteen pairing does not follow the chain direction, groove 1 (i) has no loop, groove 2 (w) has the third lateral loop, groove 3 (i) has the propeller loop and groove 4 (n) has the first lateral loop.

### Comparison between topology and loop classifications

The presence of a groove-width signature and a predominant gc pattern for each topology (Figures 9 and 10, and Table 1) shows the importance of distinguishing unambiguously between the eight topologies, as done by ASC-G4. This is the only classification that can be associated with structural characteristics by an almost one-to-one relationship. To compare it with the loop classification, we consider two 3-tetrad antiparallel-basket G4s, 143D (90) and 2MFT. Using the method described in reference (33), where, in addition to the loop type and directionality, the groove width for the lateral loops and the number of tetrads are given, the loop classification of 143D is 3(-l_w_d+l_n_), whereas for 2MFT, it is 3(d+pd). From this classification, one can deduce that both structures are made of 3 tetrads (the number before the parentheses), that for 143D, the first loop is lateral, going anticlockwise (when looking at the stem from bottom to top), and crossing a wide groove, the second is a diagonal loop, and the third is a lateral loop, going clockwise, and crossing a narrow groove. No information is given, or can be deduced, about the other two grooves. For 2MFT, the first and third loops are diagonal and the second loop is a clockwise propeller. There is no information about the groove widths. However, the presence of two diagonal loops in 2MFT means that there might be some difficulty to bind a ligand on the top or at the bottom of this G4, depending on the length of the loops, which is an important information. Despite that, there is an additional drawback of this classification, which is that nothing relates the two structures, whereas, in the unambiguous topology given by ASC-G4, they are both antiparallel-basket, from which it can be easily deduced that their strand directions are duud, and therefore, their gc pattern in the tetrads is either saas or assa, and their groove-width signature is wini, meaning that on average the first groove width is *ca* 17.3 Å, the second and fourth, *ca* 15.1 Å, and the third, *ca* 11.4 Å.

All this shows that the loop classification method is too complex and incomplete to allow a good classification of all G4s. In addition, the loop combination is not correlated to any of the structural characteristics of the G4, like the gc pattern or the groove-width signature. However, despite its drawbacks, this classification method is still interesting because it provides some useful fine information about the structure.

### The platypus G4s

Some G4s are more complex than expected, although easily described by both topology and loop classification methods. In our set, there are six one-block structures that we called platypus G4s because, like platypus, they present heterogeneous characteristics. Two of these structures, 2IDN (61) and 3QLP (62), have a 5′–5′ inversion of polarity site, meaning that their first three nts are reversed with respect to the rest of the chain. These structures have their strand directions (dudu) and loops combination (-l-l-l) corresponding to an antiparallel-chair topology, whereas their gc pattern (aasa) and their groove-width signature (iwni, see Figure 8C), are those of a hybrid1 topology. For this reason, in Figure 9, they were included in the hybrid1 groove widths, instead of the antiparallel-chair.

The four other platypus G4s are fluorogenic RNA aptamers, 7OA3, 7OAV, 7OAW and 7OAX (60). To date, they are the only one-block left-handed monomers. Their strand directions (dudd) and groove-width signatures based on C3’ atoms (wnii, Supplementary Figure S2C) correspond to hybrid3 topology, whereas the gc pattern of their two tetrads (aaaa) and groove-width signatures based on C5’ atoms (iiii, Supplementary Figure S2D) show a parallel topology, and their basic loop combinations are those of basket2 (-*l*+l+p+l).

Conversely, the interlaced dimers and monomer are not platypus G4s despite their particularity because, apart from the inserted nt coming from another chain, all their structural characteristics are similar to those of a parallel topology with a snapback or a hybrid1 topology, as for 1JJP (63).

### Classification of the stacking guanines within the strands

Based on the observation of the canonical G4s, Dvorkin *et al.* (33) classified the vertical succession of gcs along each strand in three stacking types. Type 1: when having the same gc throughout the strand (*syn-syn-syn* or *anti-anti-anti*); type 2: alternate 2–1 or 1–2, meaning that two similar gcs are followed by one different gc or one different gc is followed by two similar gcs (*syn-syn-anti*, *anti-anti-syn, anti-syn-syn*, or *syn-anti-anti*); type 3: alternate 1–1, meaning that each gc is different from the preceding one (*syn-anti-syn* or *anti-syn-anti*). Here, we extend this classification to all regular G4s. This classification concerns more precisely the 3-tetrad structures. In the 2-tetrad G4s, there are only two types, either the two gcs along the strand are similar or they are different. However, in Figure 10, there are 4-tetrad G4s, which still obey to the 3-type classification, and can be generalized as follows: type 1: only similar gcs in the strand, type 2: similar and alternate gcs in the strand, and type 3: only alternate gcs in the strand. In Figure 10, if we follow gcs along the strands (columns) instead of the tetrads (lines), we observe that type 1 seems specific to parallel structures, type 2 to hybrid structures, and type 3 to antiparallel structures. Although this is true for the majority of the structures, whatever the number of tetrads, it is not systematic. Indeed, in our set, stacking type 1 (*anti-anti* and *syn-syn*) was also found in 9% of the antiparallel structures (in the 9 antiparallel-basket2 G4-RNAs, made of only 2 tetrads) and 24% of the hybrid structures (some of them due to modified guanines, like ^F^G and oxo-G, or to mixing DNA with RNA, or to the presence of a 2-nt snapback). Stacking type 2 (*syn-anti-anti* or *anti-anti-syn*) was observed in 20% of the parallel structures (in the presence of discontinuities, like 7CLS (95) and 2MGN (10), see Supplementary Figure S3A,B, or in case all tetrad 1 is made of *syn*-G, i.e. ssss, as in 2L88 (103), 6ERL (3), 6JWD (104), 6JWE (104) and 8GP7 (105)) and 9% of the antiparallel structures (see next subsection). Contrary to the other two types, stacking type 3 was only found in the antiparallel structures. This is illustrated in Supplementary Figure S6, in the Supplementary Data.

### Ending the stacking guanines with an *anti*-G is favored over *syn*-G

Although type 3 (i.e. alternate 1–1) is systematic in the 4-tetrad antiparallel structures, it is only found in 1/3 of the 3-tetrad antiparallel G4s. The other 2/3 of the 3-tetrad antiparallel G4s are of type 2. This is probably due to the fact that the stacking of two guanines in a strand ending with a *syn*-G has higher energy than when it ends with an *anti*-G, as shown by Molecular Dynamics (MD) (106) and Quantum Mechanics (QM) (107) studies. In our set, 67% of the stacking guanines considered two by two are *anti–anti* and 25 % are *syn–anti*, whereas only 4% are *anti-syn* and 4% are *syn–syn*. Although these occurrences are biased by the number of structures of each topology type, the important difference between the occurrences shows that ending with *anti-*G is favored over ending with *syn*-G. This is also corroborated experimentally in reference (3), where the authors intended to force the presence of *syn*-Gs by modifying three or four guanines to 8-Bromo-2′-deoxyguanosines in the 3′-tetrad of a parallel *MYC* structure. It resulted in the dramatical decrease of the melting temperature by more than 40°C, and a mix of unfolded G4s with an all-*anti*-G parallel structure.

### There is no one-to-one relationship between the topology and the circular dichroism spectra

The circular dichroism (CD) spectra are often used to determine the type of topology, parallel, antiparallel, or hybrid, of a G4 with an unknown structure. This generally works well since it was described that the CD spectra of G4s are mainly due to the relative orientation of guanine bases that stack over each other in the strands (37,108), i.e. the stacking types, and each stacking type mostly corresponds to a specific topology. However, the correspondence between the topology and the CD spectrum is not systematic, mainly due to two reasons. First, the stacking of flanking nucleotides and loops also participate more or less to the CD spectra (38), depending on the tightening of their stacking. Second, as presented above and in Supplementary Figure S6, in the Supplementary Data, there are numerous exceptions to the correspondence between the stacking types and the topologies. Consequently, although the CD spectra generally indicate the right topology, this is not always the case, and there is no one-to-one correspondence between the CD spectra and the topologies. This is the case when there is a mismatch between the topology and the stacking types. For instance, when some hybrid G4s have only stacking type 1 (5MBR (109), 5MCR (109), 5OV2 (110), 6FFR (58), 6L8M (111), 6R9K (112), 6TCG (113), 7O1H (114)), their CD spectra exhibit the characteristics of parallel structures. Similarly, when parallel structures start with a top tetrad made of four *syn*-Gs (ssss), as in 6ERL (3), 6JWD (104) and 6JWE (104), i.e. with stacking type 2, the CD spectra are those of hybrid structures, and for antiparallel structures also, when having type 2 stacking, the spectra are either those of hybrid G4s (6F4Z (115) and 6YEP (116)) or those of antiparallel G4s without the trough at 260 nm, the frequency which corresponds to the peak of parallel structures (2MBJ (102), 5YEY (117), and 6JKN (118)). The other structures with mismatches between the topology and the stacking types, have no published CD spectra, except 2L88 (103), a structure similar to 6ERL, 6JWD, and 6JWE with the top tetrad ssss, and the CD spectrum of a parallel topology. On the other hand, the presence of a snapback in a G4 modifies the stacking type of two of its successive guanines, but not its CD spectrum. This could be imputable to the fact that this modification, which touches only one succession in one strand, is not strong enough to alter the spectrum. However, we will see below, in the subsection ‘Quantification of the relative orientation of the stacking stem-guanines’, that this is not the real reason.

### Reading the vertical gc succession along the strand should not follow the 5′→3′ tetrad direction, but the nucleotide chain direction

**Figure 11.**
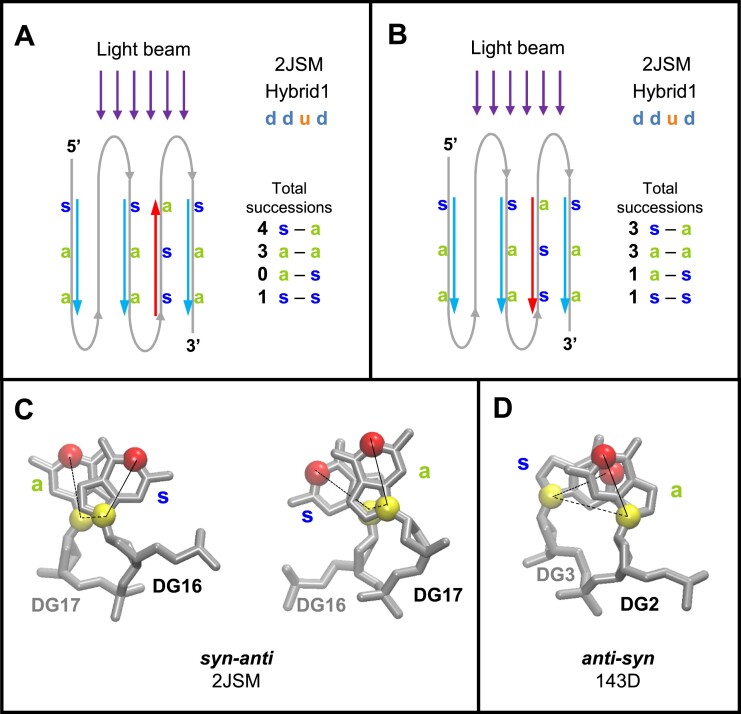
Comparison of the *syn-anti / anti-syn* successions. (A, B) A schematic representation of the telomeric hybrid1 structure 2JSM is depicted. The reading follows the nucleotide chain direction (**A**) and the 5′→3′ tetrad direction (**B**). Only the configurations of the stem guanosines are given: blue ‘s’ stands for *syn-*G and green ‘a’ for *anti-*G. The arrows indicate the directions of the succession reading. They are colored in cyan, when the succession is not ambiguous, and in red when it is. Near each structure is given the total number of each gc succession type. (**C**) The two nts of 2JSM that present ambiguity in their gc successions are depicted in two opposite directions. (**D**) An unambiguous *anti-syn* succession, taken from structure 143D. (C, D) Atoms N1 are red spheres and N9 yellow spheres. They are related with thin lines that represent the pseudo-dihedral angle Ψ.

In this study, the direction of the gc vertical succession, which is the gc progression along the strand, follows the direction of the nucleotide chain, i.e. if the strand is down the succession is read from top to bottom, and if it is up, from bottom to top (Figure 11A). Therefore, when a couple of stacking guanines ends with a *syn*-G, this *syn*-G can be in the top tetrad, if the strand is up. However, generally, in the literature (38,119,120), the gc succession is always read in the same direction, i.e. from the 5′ to the 3′ tetrad, whatever the direction of the strand. This is illustrated by the example of 2JSM (121), given by del Villar-Guerra *et al.* (38), for their comparison with CD spectra. 2JSM is a hybrid1 G4, which has strands 1, 2 and 4 downward, and strand 3 upward. The illustration of its reading in reference (38) is reproduced in (Figure 11B).

This gc succession reading is due to two considerations: (i) generally, for the stacking studies, the entire tetrad is taken into account (30), obliging to read all successions in the same direction, whereas here, we handle each strand separately; (ii) the implicit idea behind reading the stacking in the 5′→3′ tetrad direction is probably that, when experimentally the light beam hits the G4, it ‘reads’ all gc successions along the same direction of the strands. But, since the G4s tumble in the liquid solution, they are not all aligned in the light beam, and therefore, they have the same probability of being in two opposite directions. In this case, the *syn-anti* succession transforms into *anti–syn* and vice versa. So, the way of reading the gc succession should not depend on the orientation of the G4 but on an intrinsic property, like the chain direction.

We demonstrate below that the reading of the gc succession along the 5′→3′ tetrad is not appropriate but should follow the chain direction. For this, we will consider again the example of 2JSM and compare the chain-direction reading with that of reference (38). In Figure 11, for strand 3 that goes upward, when the gcs are read from top to bottom, the succession types are *anti-syn* and *syn-syn*, and from bottom to top, *syn-syn* and *syn-anti*. Here, the term ‘succession types’ qualifies every two stacking guanine bases and should not be confused with the stacking types 1, 2 and 3 that were mentioned in the previous subsection entitled ‘Classification of the stacking guanines within the strands’. There are four succession types: two homopolar, *anti–anti, syn–syn* and two heteropolar, *anti–syn, syn–anti* and here, *syn–anti* and *anti–syn* are different types, which was not the case in the previous subsection. Therefore, in strand 3 of 2JSM, the *syn-syn* succession type has no ambiguity since it is homopolar, while the heteropolar succession type, corresponding to nts DG16 (*syn*) and DG17 (*anti*), can be read in two ways. In Figure 11C, we depicted the concerned nts of 2JSM (DG16, DG17) in the two opposite directions. As expected, this did not modify their relative orientation, although it reversed their succession type. To decide to which type these stacking guanine bases correspond, we depicted in Figure 11D another structure, 143D (90), an antiparallel-chair, which starts, in its first down strand, with an unambiguous *anti–syn* succession type. As observed, the relative orientation of its bases is different from that in strand 3 of 2JSM, confirming that, whatever the orientation, the succession in 2JSM should be considered *syn–anti*, as is the case when following the direction of the nucleotide chain. Actually, this is justified by the fact that the orientations of different gcs depend on the position of G in the chain. If G*_i_*, at position *i* in the sequence, is *syn* and G*_i+1_* is *anti*, the angle between the two bases is different from the case when G*_i_* is *anti* and G*_i+1_* is *syn*, as we show in the next subsection.

### Quantification of the relative orientation of the stacking stem-guanines

To quantify the relative orientation of the stacking stem-guanines, we calculated the pseudo-dihedral angle Ψ = N1(*i*)-N9(*i*)-N9(*j*)-N1(*j*), where *i* and *j* are the identifications of the stacking guanines (for atom names N1 and N9 see Figures 11C,D). The index *j* is not necessarily equal to *i+*1. It can be very different because of the presence of discontinuities in some strands. We calculated the Ψ angles of the four succession types for the 332 G4s of our set (excluding 2MCC (50)).

**Figure 12.**
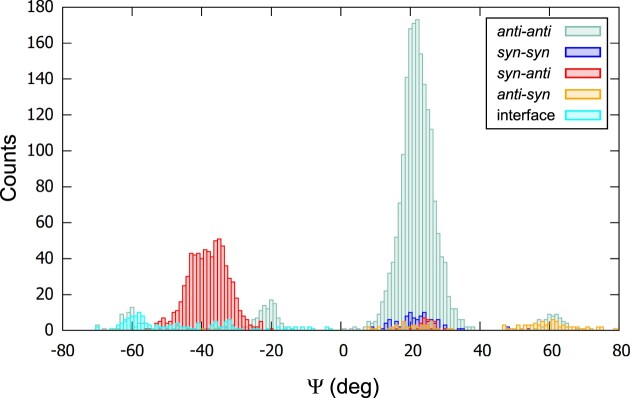
Distributions of all the gc succession types when the reading follows the nucleotide chain direction. The bin size is 1°.

*Comparison between the two ways of reading the gc successions*. First, we compared the distribution of the Ψ angles for the *syn-anti* and *anti-syn* types, when the reading follows the chain direction (Supplementary Figure S7A in the Supplementary Data) or the 5′→3′ tetrad direction (Supplementary Figure S7B). In the first case, there is a clear separation between the two populations: the *syn-anti* succession type is centered on Ψ = -40° while the *anti-syn* is centered on Ψ = 60°. In the second case, the two populations are mixed. This demonstrates that the reading of the gc succession along the strand should be done by following the chain direction, not the 5′→3′ tetrad direction. In Supplementary Figure S7A, the *syn-anti* and *anti-syn* populations around 20° correspond to successions in some parallel structures and in the G4-RNA antiparallel-basket2 structures, where three strands are homopolar and one strand is heteropolar due to a discontinuity between the two stacking guanines (like the separation between two blocks or in the presence of a long bulge or a snapback). The only ‘oddity’ concerns the angle Ψ = 92°, which corresponds to the succession in the first strand between nts DG2 and DG3 of structure 5J05 (33). In this structure, the two nts don’t stack correctly over each other because of the shortness of a 1-nt lateral loop. This is the only 1-nt lateral loop in our set. Therefore, this point is omitted in Figure 12, where the distributions of the Ψ angles are given for all succession types.

*Distribution of the Ψ angles*. Remarkably, whereas each gc succession type has only one peak, *syn–anti* centered on –40°, *syn–syn* on 20° and *anti–syn* on 60°, the *anti–anti* type presents four peaks, centered on –60°, –20°, 20° and 60°. This multiplicity of peaks is due to the presence of the two-block structures (right-handed and left-handed) plus the five one-block left-handed structures, which all consist of about 91% of *anti-anti* stacking. We observe a rule that applies to the *anti-anti* successions in both one-block and two-block structures: generally, within a block, if the twist between two successive tetrads is right-handed, the Ψ angles are around 20°, and if it is left-handed, they are around –20°. There are however two exceptions to this rule. The first exception concerns the four platypus fluorogenic RNA aptamers, 7OA3, 7OAV, 7OAW and 7OAX (60). Although these structures are one-block left-handed, they have all their Ψ angles around 20°, instead of –20°. The second exception concerns some hybrid4 structures. In the hybrid4 structures, considering that the gc pattern in tetrad 2 is always saaa and the direction of strand 4 is up there are three possibilities concerning the succession type in strand 4, between tetrads 3 and 2: (i) *syn-anti*, when the gc pattern of tetrad 3 is asss, like in Figure 10; in this case, the Ψ angle was found around –40°, as expected; (ii) *anti–anti*, when the gc pattern of tetrad 3 is saaa, like in Supplementary Figure S6, bottom of the first column; in this case, the Ψ angle was found around 20°, still as expected; (iii) *anti–anti*, when the gc pattern of tetrad 3 is of the unusual form, assa, which is sometimes observed in the presence of a 5′-bottom snapback in strand 1, as discussed above and illustrated in Supplementary Figure S3D; in this case, the Ψ angle was found around –40°, not as expected for the *anti–anti* succession type, but like the angle of the *syn–anti* type, which was expected if the gc pattern of tetrad 3 were of the usual form, which is asss. Therefore, interestingly, even if the guanosine located at the junction between tetrad 3 and strand 4 is clearly an *anti*-G, it is oriented relative to its above guanosine as if it were *syn*, as expected for the asss pattern which is coherent with the strand directions of hybrid4, duuu.

Apart from this particular case of hybrid4 structures, all regular structures have the Ψ angles of their *anti-anti* successions within the peak around 20°, even in the presence of snapbacks. There is one interesting exception in structure 6JCD (93), a hybrid2 G4, where the presence of an *anti-*G snapback yields an *anti-anti* Ψ angle in the peak around –40°, which corresponds to a *syn-anti* succession. Remarkably, in a 2-tetrad hybrid2 structure, like 6JCD, a *syn–anti* succession is expected in strand 2 between tetrads 1 and 2 (see Figure 10), instead of what, in 6JCD, it is replaced with an *anti–anti* succession because of the snapback, but with a conserved *syn–anti* Ψ angle. This is also the case of the *syn-anti* or *anti-syn* successions due to snapbacks in parallel structures, where the Ψ angle is that of an *anti–anti* succession, as expected in parallel G4s. This means that, in the presence of a discontinuity, although the gc succession is modified (because of the reversed micro-strand direction, as shown in Supplementary Figure S3), the Ψ angle is conserved. This explains why the snapbacks do not modify the CD spectra of G4s. In addition to that, in the left-handed parallel G4 structures, where all Ψ angles are around -20°, the CD spectra are inverted with respect to the right-handed parallel G4s, where all Ψ angles are around 20° (59).

Our calculations show that the relative orientations of the guanines (i.e. the Ψ angles) between two tetrads, are the same for the four strands. Therefore, even if, for some reason, like the inversion of the micro-direction of the strand, the gc of one guanine is modified, the Ψ angle between this guanine and its stacking partner should keep the same value as for the other three stacking couples. This is consistent with the classification of the stacking tetrads proposed in references (30,119) where three standard stacking modes of two successive tetrads are given: same partial 5/6-ring (S5/6) mode, opposite 5-ring (O5) mode, and opposite partial 6-ring (O6) mode, corresponding to the *anti-anti, syn-anti*, and *anti-syn* successions, respectively. However, this is not applicable to the peaks of Ψ at –20°, –60° and 60° (for *anti-anti*), which are observed in Figure 12, and concern the two-block structures.

Considering the two-block structures, the stacking within each block obeys to the same rule, i.e. Ψ is around 20° for the right-handed twist and around –20° for the left-handed twist. This is usually true because these structures consist of parallel blocks (when the block is made of 2 tetrads), which mostly consist of *anti*-Gs. However, 7MKT (122), which is a 3-tetrad left-handed two-block structure with the topology parallel/−, is an exception because its first block consists of all *syn*-Gs. This is the only structure in our set with two consecutive tetrads entirely made of *syn*-Gs, and therefore, all its Ψ angles are around 50°. Consequently, we complete the above rule: within an all *anti*-G block, Ψ is around 20° for the right-handed twist and around -20° for the left-handed twist, and within an all *syn*-G block, Ψ is around 50° for the left-handed twist. Nothing can be concluded about the all *syn*-G right-handed block because there is no such structure in our set, and the Ψ angles of the *syn–syn* successions in Figure 12 cannot be of any help. Indeed, the *syn–syn* couples in the histogram correspond to the succession in one strand and, according to the gc patterns, the other three strands are always *anti-anti*. As presented above, the minority *syn-syn* strand will adopt the Ψ angles of the majority *anti–anti* strands. This may explain why their Ψ angles are around 20°, like *anti–anti* in regular G4s.

Regarding the separation between the two blocks, there is no strict rule (Supplementary Table S4 in the Supplementary Data), despite a difference observed between the parallel/parallel structures and the others. In the parallel/parallel structures, when the relative twist of the two blocks is right-handed, the Ψ angles are around 20°, and when left-handed, around -60°. In the parallel/hybrid2 structure, the separation is right handed with Ψ around –20°. In the −/parallel structures, where block 1 is made of one tetrad, either the separation is left- or right-handed, Ψ is around 20°. Finally, in the parallel/− structures, where block 2 is made of one tetrad, the separation is right-handed with Ψ, either around 20° or 60°. The parallel/− are the only structures of our set with *anti-anti* successions that populate the distribution of Ψ around 60°.

*Interface between the monomers in the stacking-stem dimers*. Thirty-three of the structures in our set form stacking-stem dimers, twenty-five are one-block G4s and eight, two-block G4s. They are all parallel, made of *anti*-Gs. The dimer stacking is generally top-to-top, except for the one-block left-handed structure, 6FQ2 (59), and the two-block right-handed structures 8EYU (123), 8EYV (123), 8EYW (123) and 8F0N (123), where it is tail-to-tail. In the tail-to-tail two-block structures, –20° < Ψ < 0°, whereas in the seven interlaced dimers, which are right-handed G4s, –40° < Ψ < –20°. For all the other structures, the distribution of the Ψ angles presents two small peaks, around –60°, mostly for left-handed interface, and –45°, mostly for right-handed interface, without a clear separation between the two groups.

### Relationship between the twist and tilt angles and the Ψ angle

**Figure 13.**
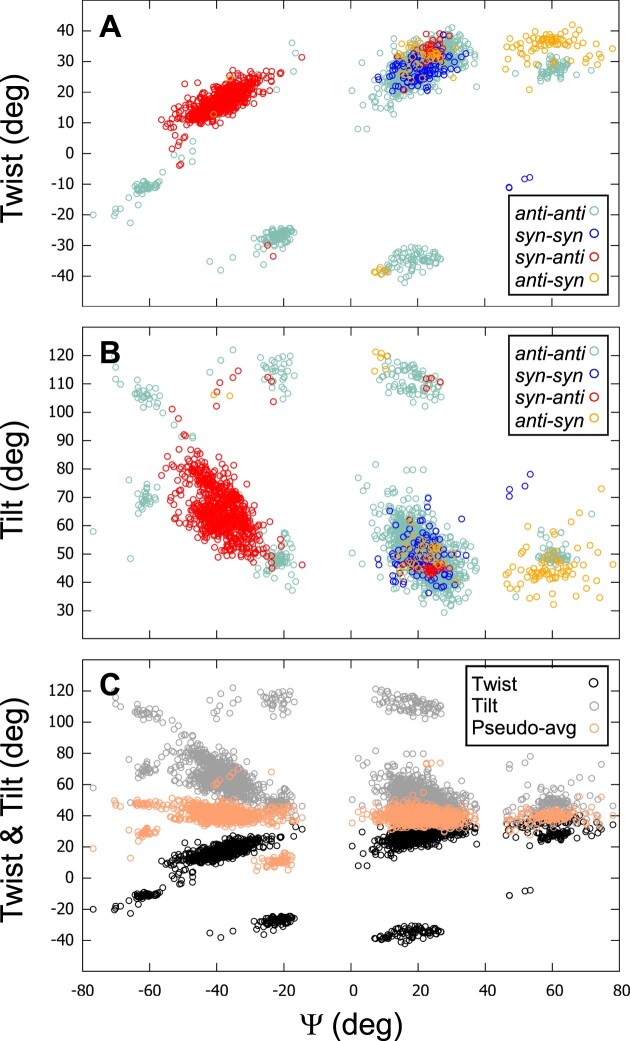
Twist and tilt angles *vs* Ψ angle. **(A, B)** The dots are colored according to the gc succession types: pastel teal for *anti-anti*, blue for *syn–syn*, red for *syn–anti*, and orange for *anti–syn*. The reading of the gc successions follows the nucleotide chain direction. (**C**) The same twist (black) and tilt (gray) angles are reported versus Ψ as well as their pseudo-average (light salmon).

As observed above, the relative orientation of the bases in the stem depends on both the gc succession and the handedness (obtained from the twist angles). Therefore, we reported in Figure 13 the twist and tilt angles *versus* the Ψ angle. For the definition of the twist and tilt angles see reference (31). In Figure 13A,B, we colored the points according to the gc succession, when read along the chain direction. We observe that the five peaks of the Ψ angle distributions of Figure 12, excluding the stacking-stem dimer interface, are split here into mainly six distinct packets: three packets for the right-handed twist, i.e. with positive twist angles, and mainly with acute tilt angles, and three packets for the left-handed twist, i.e. with negative twist angles, and mainly obtuse tilt angles. In addition to that, there is an isolated small packet, which consists of four points, with negative twist angles (left-handed) but with acute tilt angles. These points correspond to the four *syn–syn* successions in the first block of the special structure, 7MKT (122), whose sequence is (GU)_11_G.

The packets are better delimited in the twist than in the tilt angles. The absolute value of the twist angle increases with increasing Ψ, while this progression is reversed for the tilt angle. The few *syn–anti* and *anti–syn* negative twist angles correspond to the *syn*–Gs present in some left-handed G4s, where they behave like *anti-*G. For the regular G4s, the twist and tilt angles are correlated to the Ψ angles with the correlation coefficients of 0.87 and -0.70, respectively (*p*-values < 10^−15^), and the twist and tilt angles are anticorrelated (*R =* –0.72, *p*-value < 10^−15^). Since the Ψ angles reflect the gc succession types, excluding some discontinuities, this means that the twist and tilt angles are correlated to the gc successions along the strands. The average twist and tilt angles for each gc succession type in the regular G4s is given in Table 3.

**Table 3.** The average twist and tilt angles for each gc succession type in the regular G4s

	*syn–anti*	*syn–syn*	*anti–anti*	*anti–syn*
Twist angle	17.4 ± 4.9°	27.7 ± 3.7°	28.8 ± 3.7°	34.0 ± 4.3°
Tilt angle	64.5 ± 9.0°	49.3 ± 6.6°	49.8 ± 6.3°	46.4 ± 8.4°

### The G4 four helices twist similarly and sometimes irregularly

The values of the twist angles and their dependency on the gc succession constitute an additional demonstration that the reading of this succession should be done along the chain direction. Indeed, the average twist angle for the *anti–syn* type (∼34°) is twice bigger than that of the *syn–anti* type (∼17°). If we consider again the example of 2JSM (121) (Figure 11A,B), when reading the gc succession between tetrad 1 and tetrad 2, along the chain direction, the four strands show the succession *syn–anti*, meaning that the twist between the two tetrads is about 17°. Contrarily, when reading it along the 5′→3′ tetrad direction, strand 3 has the succession *anti–syn*, with an angle ∼34°, whereas the other three strands are *syn–anti*, with an angle ∼17°. In the latter case, the G4 would not be stable since the twist of one tetrad with respect to its preceding one would correspond to one angle (∼17°) for the ¾ of the tetrad (three strands) and twice this angle (∼34°) for the other ¼ of the tetrad (one strand). Therefore, when reading the gc succession along the chain direction, the twist between two successive tetrads of all four helices is the same. This is even the case when the gc succession is *anti–anti* in three strands and *syn–syn* in one strand, as is the case, for instance, between tetrads 2 and 3 in hybrid 1, 2, and 3 structures in Figure 10, because the two different gc successions correspond to similar Ψ angles, about 20° and therefore to similar average twist angles, about 28° (Table 3).

However, along each helix, the twist angle can be different between every two tetrads, making them irregular helices. For instance, if we consider the gc pattern of a 4-tetrad antiparallel-basket G4, as given in Figure 10, the direction of its strands is duud, and the gc progression in the tetrads is saas for tetrads 1 and 3 and assa for tetrads 2 and 4. Therefore its gc successions along the four strands, when read in the chain directions, are all *syn-anti* for tetrads 1–2, and 2–3 and *anti-syn* for tetrads 2–3. Consequently, all four helices (or strands) have a twist angle between tetrads 1–2, and 2–3 about 17° and between tetrads 2–3, about 34°. This makes all four helices similar but irregular. A similar reasoning holds for the tilt angles.

### The pseudo-average of the twist and tilt angles is constant

Since the progression of the tilt and twist angles with respect to Ψ takes place in opposite directions, we reported these angles in Figure 13C and calculated their pseudo-average, meaning that the twist angle was added to the tilt angle and the sum was divided by two. As expected, this pseudo-average is approximately constant, with a mean value about 40°. The corresponding points form a cloud along a horizontal line, which seems to be an asymptote to both the twist and tilt angles. However, we observe two groups of points which are not on this line. (i) The pseudo-averages above 60°, for Ψ between –20° and –40° correspond to the two platypus structures, 2IDN (61) and 3QLP (62), and (ii) the pseudo-averages below 20° correspond to six left-handed structures, 6FQ2 (59), 6GZ6 (59), 7D5D (124), 7D5E (124), 7D5F (124) and 7DFY (125). These eight structures have the particularity that their first stem-guanine, an *anti*-G, is oriented opposite to usual (Supplementary Figure S1A), meaning that its hydrogen-bond donors are oriented to the right (like a *syn*-G) rather than to the left (like a usual *anti-*G), when looking at the stem from top to bottom. Therefore, the strand numbering of these structures is exceptionally anticlockwise, and consequently, their tilt angles are the supplementary angles to those of a structure with the right orientation of the *anti*-G, which explains the difference with the other pseudo-averages. However, to investigate if they still obey to the same rule of the constant pseudo-average, we renumbered the strands of these structures in the clockwise orientation and carried out the calculation of their tilt angles. The resulting pseudo-averages were then around 40° like the others. Interestingly, this shows that the pseudo-average of the twist and tilt angles constitutes an easy way to identify the structures with a reversed first stem-guanine without visualizing them.

The eight structures mentioned above are the only G4s of our set to have their first stem-guanine in the opposite orientation to usual. 2IDN and 3QLP are right-handed helices. The opposite orientation of their first stem-guanine is due to the 5′-5′ inversion of polarity site. 6FQ2 (a dimer of one-block G4), 6GZ6, 7D5D, 7D5E, 7D5F and 7DFY (five monomer two-block G4s) are left-handed helices (see Supplementary Table S4 in the Supplementary Data), in which the first stem-guanine is located in tetrad 1. However, all the left-handed G4s do not have their first stem-guanine in the opposite orientation, even when it is located in the left-handed part. In our set, there are six other all left-handed G4s: the two-block structures 2MS9 (28) and 4U5M (28), and the four one-block platypus structures 7OA3, 7OAV, 7OAW and 7OAX (60). The two G4s, 2MS9 and 4U5M, have the same sequence and they share between 54% and 90% of the sequence of the 5 two-block structures (6GZ6, 7D5D, 7D5E, 7D5F, and 7DFY). Despite that, their strand numbering is clockwise because their first stem-guanine is *syn*. Probably, this *syn* configuration is due to the presence of a 5′-flanking nucleotide. Regarding the four platypus structures, their first stem-guanine is a well-oriented *anti*-G, probably because of the presence of eleven 5′-FNs of which eight form a right-handed dsRNA with eight of the eleven 3′-FNs. Conversely, in the six left-handed G4s with the anticlockwise orientation (6FQ2, 6GZ6, 7D5D, 7D5E, 7D5F and 7DFY) there are no 5′-FNs. Therefore, it seems that, when the first stem-guanine is part of a left-handed helix, and in the absence of any 5′-FNs, its orientation is opposite to usual, leading to the supplementary tilt angle, whereas, in the presence of at least one 5′-FN, the first stem-guanine adopts the usual orientation, and therefore, the tilt angle and the pseudo-average of tilt and twist angles are right.

### Relationship between the twist and tilt angles and the minimum C3’–C3’ distances for the regular G4s

The twist of the tetrads, which is dependent on the vertical gc succession, results in the tilt of the strands, which impacts the space available in the grooves. This space is generally different from the groove width. Indeed, the groove width is the average of the distances C3’–C3’, C5’–C5’, or P–P, which are calculated, for each groove, between the facing guanosines in the same tetrad (Figure S8A in the Supplementary Data), whereas, due to the tilt of the strands (Supplementary Figure S8B), the distances C3’–C3’, C5’–C5’, or P-P, between guanosines from different tetrads may be smaller (Supplementary Figure S8C). This is called the minimum groove distances in ASC-G4. For each tetrad, a minimum groove distance is calculated. However, due to the acute tilt angles in the regular G4s, the minimum distance of the first tetrad is mostly equal to the distance of the facing Hbps of guanosines. Therefore, we discarded this tetrad from our calculations, and only considered the minimum distances for each of the other tetrads. The minimum distances represent better the real space available in the groove than the usual distances.

**Figure 14.**
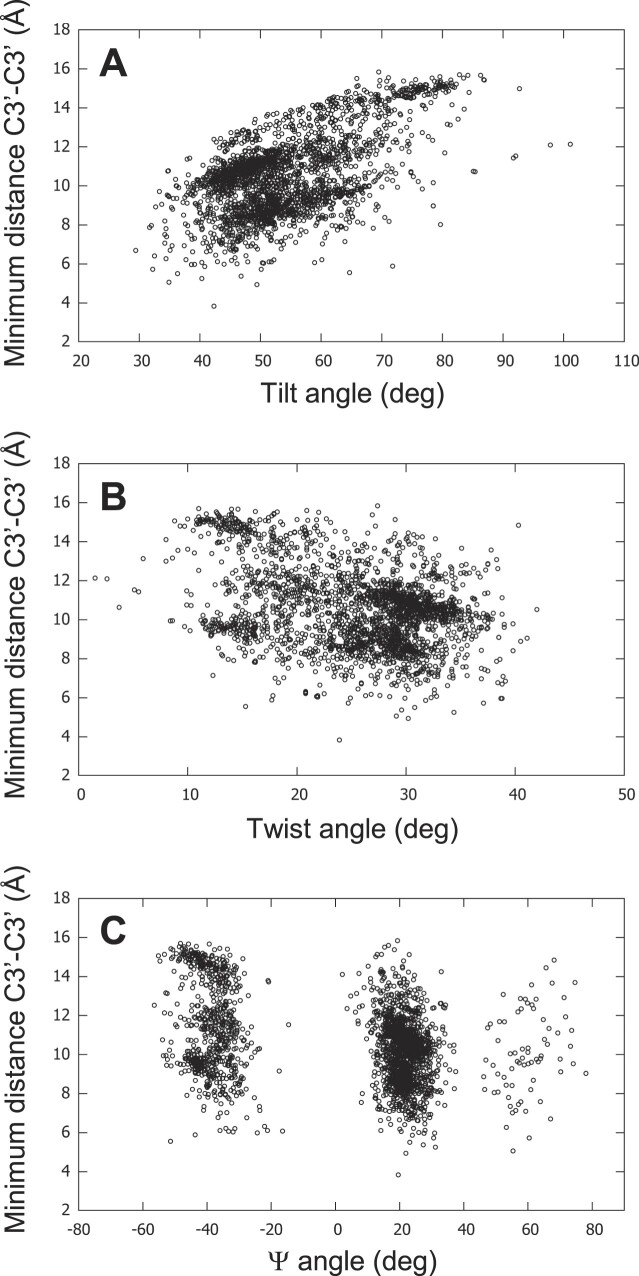
Minimum C3’–C3’ distances versus tilt (**A**), twist (**B**), and Ψ (**C**) angles for regular G4s. Since the three angles are calculated between tetrads *i* and *i +*1, the minimum distances are those of the guanosines in tetrad *i +*1.

To investigate the impact of the tilt on the minimum groove distances in regular G4s, we reported in Supplementary Figure S8D in the Supplementary Data the minimum C5’–C5’ distances *vs* the tilt angles. The correlation between these two quantities (0.46) is small, although significant (*p*-value < 10^−15^). However, the range of the minimum C5’–C5’ distances is between 5.0 and 17.8 Å, which is greater than the range of the minimum C3’–C3’ distances, which is between 3.8 and 15.9 Å. Therefore, the latter was preferred because it is smaller and hence it represents better the space available within the groove. The minimum C3’–C3’ distances are reported *vs* the tilt, twist, and Ψ angles in Figure 14. Their correlation with the tilt angles (0.57, *p*-value < 10^−15^) is greater than that calculated for the C5’ atoms, probably because atom C3’ is united to the core of the guanosine, with less freedom of movement than atom C5’. There are also little, although significant, anticorrelations of the minimum C3’-C3’ distance with the twist angle (*R* = –0.35, *p*-value < 10^-15^), and the Ψ angle (*R =* –0.34, *p*-value < 10^−15^). From this, we conclude that it is mainly the tilt angle that is responsible for the shorter distances between the C3’ atoms that delimit the groove.

We also investigated if there is any relationship between the loop types or lengths and the minimum groove width, but we could find none, neither by considering atoms C3’ nor atoms C5’. Therefore, we concluded that only the vertical gc succession along the strands determines the twist and tilt angles, which in turn impact the minimum groove widths.

*Recommendations for lateral ligand binding*. Generally, the space available to accommodate a ligand is smaller than the groove width given above. The difference between individual distances and minimum distances may go up to 9.1 Å for regular structures. In some cases, C3’ atoms from adjacent strands in medium and narrow grooves are in close contact, with minimum C3’–C3’ distances below 6 Å. Therefore, for the design of lateral ligands, it is important to consider the minimum groove width rather than the groove width. Besides, some grooves are obstructed with propeller loops. This is the case of three over the four medium grooves in parallel structures, and the narrow grooves in most hybrid4 structures, which are obstructed with 0-nt propeller loops. However, despite the smallness of their minimum groove widths and the obstruction of their loops, a few parallel structures, with ligands bound on the top or at the bottom of the stem, seem to have some parts of the ligand that also bind in the grooves, as suggested, for instance, for structures 3CDM (126) and 3SC8 (84), in Figure 1A of reference (127). But, scrutinizing these structures shows that this is not the case (Supplementary Figure S9 in the Supplementary Data). The concerned parts of the ligands are positioned on a layer of water molecules within the groove, with no direct interaction with the groove nts, or very little. This means that they do not bind to the groove, although, in these cases, the propeller loop is long enough (3 nts) to get aside. Based on these considerations, we recommend avoiding targeting parallel structures with lateral ligands, and privilege targeting with such ligands preferentially wide grooves, which, in addition, are never obstructed with a propeller loop.

## Conclusions

The study of all the intramolecular G4 structures available to date using ASC-G4 allowed us to clarify and formalize several characteristics and propose new notions. Usually, in the literature, three types of groove widths are described, based on the gcs of adjacent guanosines in the tetrads, narrow, medium, and wide. The quantification of the distances between these guanosines showed the presence of four types of groove widths based on gcs, instead of three: narrow, medium-narrow, medium-wide, and wide. However, the three known types of groove widths were found and quantified based on the strand directions, although these directions are strongly dependent on the gc progression of adjacent guanosines in the tetrads. This dependency explains the presence of a predominant gc pattern and a groove-width signature for each of the eight distinguishable topologies. This dependency also allows deducing the topology of regular G4s from only one tetrad, provided this tetrad does not contain any strand discontinuities, like long bulges and snapbacks. Indeed, in the presence of strand discontinuities, the gc progression is not dependent anymore on the strand direction, but rather on the strand micro-directions, which impact the gc pattern but not the groove-width signature. Because of the robustness of this signature, binding a ligand or a protein above or below the G4 stem does not modify significantly its groove widths when there is no modification of the topology.

Conversely, one cannot deduce the topology from only one strand because in the gc patterns, for each topology, there can be various gc stacking types. This explains why some published CD spectra do not present the characteristics corresponding to the G4 topology. To quantify the relative orientation of the stacking guanines, we defined a pseudo-dihedral angle Ψ. In regular G4s there are four distributions of Ψ, depending on the vertical gc succession of the guanines along the strands. In the two-block G4s and the left-handed G4s, there are five distinct distributions, four are almost all made of *anti*-Gs (91%) and one is made of all *syn-*Gs. We demonstrated that the gc vertical succession should be read following the chain direction, not the stem direction, as usually done, to avoid mixing the distributions of the Ψ angles. Besides, in regular G4s, the Ψ angle determines the helix twist, and the tilt of the strands, which impacts the minimum groove widths, reducing the available accessible space within the grooves. The reduced space in narrow and medium grooves makes lateral ligand binding in these grooves unlikely. Consequently, the design of lateral ligands targeting parallel structures may not succeed because these structures are the only ones devoid of wide grooves. Besides, the helix twist can be different for two successive pairs of tetrads, whereas, for each pair, it is the same for all four helices. We therefore, concluded that the G4 four helices are similar but irregular.

To summarize the observed rules: the horizontal gc progression in the tetrads determines the strand direction and therefore, the topology and the groove widths, whereas, the vertical gc succession in the strands determines the twist and tilt angles and therefore, the minimum groove widths, which is the real available space in the groove.

We also introduced the concept of platypus G4s, which are G4s that present characteristics from various topologies and therefore do not follow correctly the rules. In our set, there are 6 platypus G4s.

## Supplementary Material

gkae182_Supplemental_Files

## Data Availability

The calculated characteristics of all the 333 G4 structures are available in the Supplementary Data (file ASC-G4_outputs.tar.gz).

## Notes

Present address: Marc Farag, Normandie Université, Unicaen, CERMN, F-14000 Caen, France.
