# Supplementary material for: Comprehensive analysis of intramolecular G-quadruplex structures: furthering the understanding of their formalism
Source: Nucleic Acids Res. 2024 Mar 21;52(7):3522–46. doi: 10.1093/nar/gkae182 (PMC11039995; doi:10.1093/nar/gkae182)
Supplement: gkae182_Supplemental_Files [file gkae182_supplemental_files.zip › Supplementary_Data.pdf]

SUPPLEMENTARY DATA

Comprehensive analysis of intramolecular G-quadruplex structures: furthering the understanding of their formalism

Marc Farag and Liliane Mouawad*

CONTENTS

p. 2 Figure S1. The strand numbering.

p. 3 Table S1. Distribution of the topologies of the 194 one-block and interlaced structures.

p. 4 Table S2. Distribution of the topologies of the 16 two-block structures.

p. 5 Table S3. The average groove widths (Å) calculated from atoms C5', C3', and P.

p. 6 Figure S2. The groove width signature of four structures excluded from Figure 9.

p. 7 Figure S3. Example of three parallel structures with a discontinuity in a strand.

p. 8 Figure S4. Determination of the topology of a regular G4 from only one tetrad.

p. 9 Figure S5. Comparison of the dimer 1MYQ and the two-block G4 1OZ8.

p. 10 Figure S6. Classification of the stacking types of regular structures, the exceptions presenting mismatches between the topologies and the stacking types, and the incidence of these mismatches on the CD spectra.

p. 11 Figure S7. Comparison of the distributions of the *syn-anti* / *anti-syn* succession types.

p. 12 Table S4. Characteristics of the two-block structures: topology, strand directions, handedness, and center of the range of the corresponding Ψ angles.

p. 13 Figure S8. Minimum distances and tilt angles in regular G4s.

p. 14 Figure S9. Two parallel structures with a part of the ligand that seems to bind in the groove.

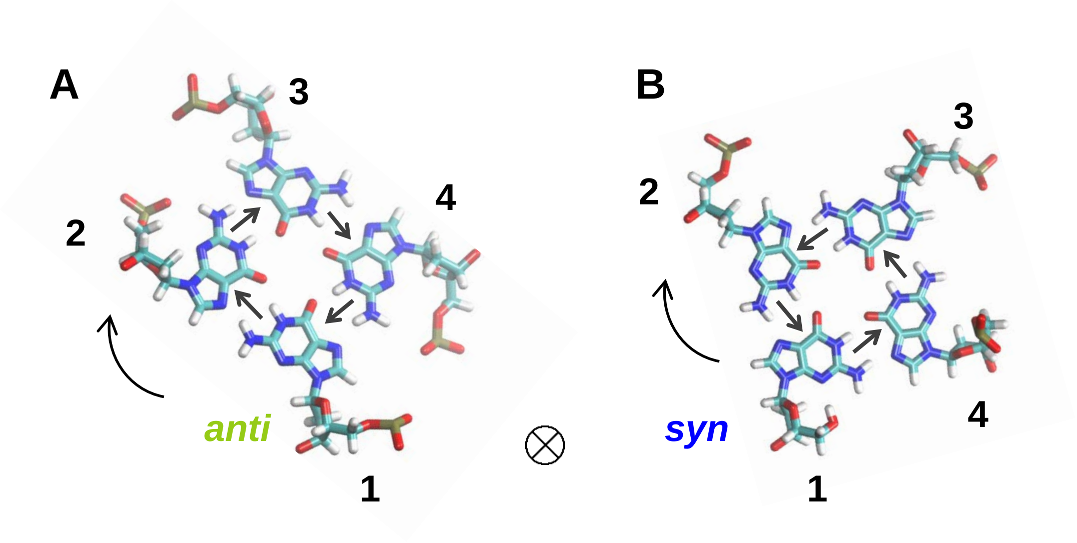

⊗ Top-to-bottom direction

Figure S1. The strand numbering. Top view of two tetrads, one starting with an *anti*-G (A), and one with a *syn*-G (B). In (A) and (B), the small straight arrows indicate the orientation of the H-bond donors in the Hoogsteen pairing. The big curved arrows indicate the orientation of the strand numbering. The strand numbers are in bold. The atoms' color code is the following: C (cyan), N (blue), O (red), P (tan), and H (white).

The numbering is done as follows: if the first stem-guanine is *anti*, the second strand faces its Watson-Crick edge, and if the first stem-guanine is *syn*, the second strand faces its Hoogsteen edge. The first stem-guanine is at the junction of the leading strand and generally the first tetrad of this leading strand. In the one-block structures, the leading strand is the first one, and therefore, the first-stem guanine is generally at the junction of strand 1 and tetrad 1, except in the presence of a snapback in the first strand, in which case, the first-stem guanine is at the junction of strand 1 and tetrad 2. In the two-block structures, strand 1 is divided in two parts, and the part that contains the lowest guanine identification (in the absence of a snapback) is the leading strand. Therefore, the first stem guanine belongs to this leading strand and the first tetrad of this leading strand.

Table S1. Distribution of the topologies of the 305 one-block and interlaced structures. The G4-RNAs are in bold, the hybrid G4-DNA-RNA structure, 6FFR, is in slanted bold and the erroneous G4-DNA structure 2MCC is in gray.

Topology	Number of structures	PDB ID
Parallel	138	1KF1, **1MY9**[D], 1MYQ[D], 1XAV, 1Y8D[I], 2A5P[3'b], 2A5R[3'b], 2KQG, 2KQH, 2KYP, 2KZE, 2L7V, 2L88, 2LBY, 2LD8, 2LE6[D], 2LEE, 2LK7, 2LPW, 2LXQ, 2LXV[D], 2M27, 2M27, 2M4P, 2M53, 2M90[5't], 2M92[3'b], 2M93, 2MB2, 2MB4[D,5'b], 2MGN[3'b], 2N21, 2N4Y, 2N60, 2N6C[3'b], 2O3M[3'b], **2RQJ**[D], **2RSK**[D], **2RU7**[D], 3CDM, 3QXR[3'b], 3R6R, 3SC8, 3T5E, 3UYH, 4DA3, 4DAQ, 4FXM, 4G0F, 4WO2[D,3'b], 4WO3[D,3'b], **5BJO**, **5BJP**, 5CCW, 5DWW[D], 5DWX, 5I2V, 5LIG, 5NYS, 5NYT, 5NYU, 5UA3[D], 5VHE, 5W77, 6AU4, **6E80**, **6E81**, **6E84**, 6ERL, 6FQ2[D,L,3't,5'b], 6H5R, 6IP3, 6IP7, 6ISW, 6JJ0, 6JWD, 6JWE, 6K3X[A], 6K3Y, 6LDM, 6LNZ, 6N65[D], 6NEB, 6O2L, 6P45[D], 6PNK, **6Q6R**[D], 6SUU[3'b], 6T2G, 6T51, 6V0L, 6W9P, 6WCK[D], 6XCL, 6YY4, 6ZL2, 6ZL9, 6ZRM, 6ZTE[3'b], 7CLS,7E5P, 7JKU, 7KBV, 7KBW, 7KLP, 7LL0, 7MSV[A], 7N7D, 7N7E, 7NWD, 7OAR, 7PNE[3'b], 7PNG[3'b], 7PNL, **7PS8**, **7Q48**, **7Q6L**, **7QA2**, 7QVQ, **7SXP**, 7WGW, 7X7G[I], 7X8M, 7X8N, 7X8O, 7XDH[I], 7XH9[I], 7XHD[I], 7XIE[I], 7ZEM[3'b], 8ABD[3'b], 8D78, 8D79, 8DUT, 8EBO, 8EDP, 8GP7, 8JFQ[3'b]
Antiparallel-chair	63	148D, 1BUB, 1C32, 1C34, 1C35, 1C38, 1HAO, 1HAP, 1HUT, 1QDF, 1QDH, 1RDE, 2IDN, 2KM3, 2LYG, 2M8Z, 2N2D, 3QLP, 4DIH, 4DII, 4LZ1, 4LZ4, 4NI7, 4NI9, 5CMX, 5EW1, 5EW2, 5MJX, 5OPH, 5YEY, 6EO6, 6EO7, 6EVV, 6FC9, 6GH0, 6GN7, 6JKN, 6Z8V, 6Z8W, 6Z8X, 7CV4, 7D31, 7D32, 7D33, 7NTU, 7OTB, 7V3T, 7W9N, 7Z9L, 7ZKL, 7ZKM, 7ZKN, 7ZKO, 8ABN, 8BW5, 8FHV, 8FHX, 8FHZ, 8FI0, 8FI1, 8FI2, 8FI3, 8FI8
Antiparallel-basket	22	143D, 1I34, 201D, 230D, 2KF7, 2KF8, 2KKA, 2M6V, 2M6W, 2M91, 2MCC[RL], 2MCO, 2MFT, 5J05, 5J4P, 5J4W, 5J6U, 5LQG, 6GZN, 6ZX6, 6ZX7, 7OQT
Antiparallel-basket2	15	2KOW, 2MBJ, **4KZD**, **4KZE**, **4Q9Q**, **4Q9R**, 5LQH, **5OB3**, **6B14**, **6B3K**, 6F4Z, 6FTU, 6YEP, **7L0Z**, **7ZJ4**
Hybrid1	16	1JJP[I], 2E4I, 2GKU, 2HY9, 2JSK, 2JSM, 2MAY, 2MB3, 2MWZ, 5MBR, 5Z80, 5Z8F, 6IA4, 6KFI, 6XT7[5't], 7CV3
Hybrid2	14	2LOD, 5MCR, 5MTA, 5MTG, 5OV2, **6E8S**, **6E8T**, **6E8U**, ***6FFR***, 6JCD[3't], 6L92, **6PQ7**, **6UP0**, 7OLH
Hybrid3	21	186D, 2F8U, 2JPZ, 2JSL, 2JSQ, 2KZD, 2MFU, 5MVB, 6AC7, 6CCW, 6IA0, 6KFJ, 7ALU, 7EL7, **7OA3**[L,5't], **7OAV**[L,5't], **7OAW**[L,5't], **7OAX**[L,5't], 7X2Z, 7X3A, 8IJC
Hybrid4	16	2KPR[5'b], 5O4D[5'b], 5ZEV[5'b], 6H1K[5'b], 6L8M[5'b], 6R9K, 6R9L, 6RS3[5'b], 6TC8[5'b], 6TCG[5'b], 6YCV[5'b], 7ATZ[5'b], 7YS5[5'b], 7YS7, 7ZEK[3't], 7ZEO[3't]

[L] Left-handed helix
[RL] Hybrid Right-handed / Left-handed helix
[D] Stacking-stem dimer
[I] Interlaced dimer
[A] Monomer with an additional G to complete the tetrad

[3'b] Structure with a 3'-bottom snapback
[3't] Structure with a 3'-top snapback
[5'b] Structure with a 5'-bottom snapback
[5't] Structure with a 5'-top snapback

Table S2. Distribution of the topologies of the 28 two-block structures. The topology and the direction of the strands correspond to Block 1 / Block2. The block that contains the first stem-guanine has its first strand colored in red. The G4-RNAs are in bold.

Topology	Number of structures	PDB ID
Parallel / Parallel<br>dddd / dddd	1	2N3M
Parallel / Parallel<br>dddd / dddd	2	6JCE[RL,3't], 6QJO[RL,3't]
Parallel / Parallel<br>dddd / uuuu	7	2MS9[L,3'b], 4U5M[L,3'b], 6GZ6[L,3'b,5'b], 7D5D[L,3'b], 7D5E[L,3'b], 7D5F[L,3'b], 7DFY[L]
Parallel / Parallel<br>uuuu / dddd	2	1OZ8[RL], **6K84**
Parallel / Hybrid2<br>uuuu / dddu	1	6KVB[3't,5'b]
− / Parallel<br>one tetrad / dddd	4	**2LA5**[RL], **5DE5**[RL], **5DE8**[RL], **5DEA**[RL]
− / Parallel<br>one tetrad / uuuu	4	**8EYU**[D], **8EYV**[D], **8EYW**[D], **8F0N**[D]
Parallel / −<br>dddd / one tetrad	7	**5V3F**[D], **6C63**[D], **6C64**[D], **6C65**[D], **6V9B**, **6V9D**, **7MKT**[RL]

[L] Left-handed helix
[RL] Hybrid Right-handed / Left-handed helix
[D] Stacking-stem dimer

[3'b] Structure with a 3'-bottom snapback
[3't] Structure with a 3'-top snapback
[5'b] Structure with a 5'-bottom snapback

Table S3. The average groove widths (Å) calculated from atoms C5', C3', and P.

	Narrow	Medium	Wide
Atom C5'	11.4 ± 0.8	15.1 ± 0.7	17.3 ± 0.5
Atom C3'	12.9 ± 0.5	14.4 ± 0.5	15.6 ± 0.3
Atom P	9.4 ± 1.3	16.0 ± 0.9	20.0 ± 0.7

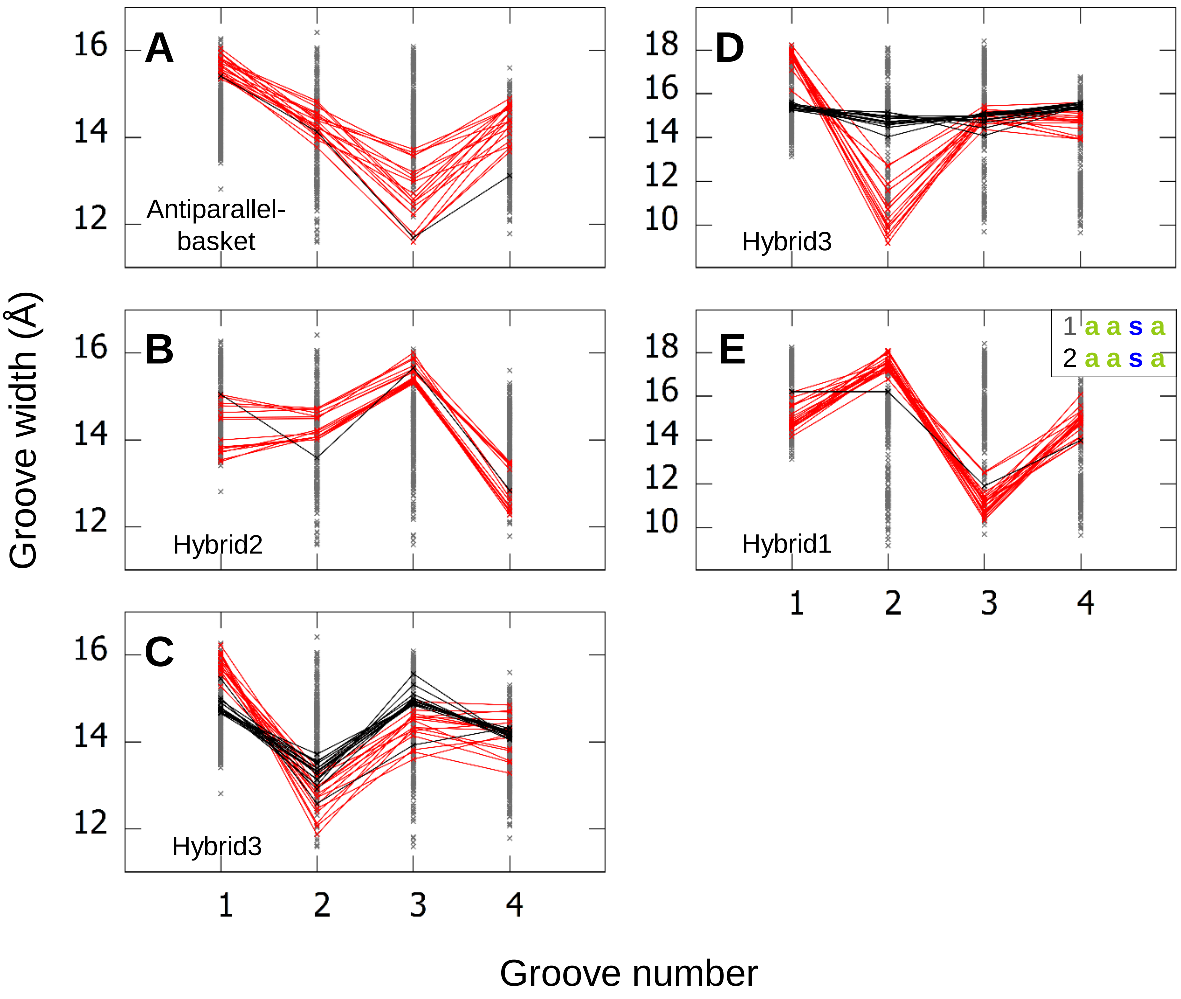

Figure S2. The groove-width signature of eight structures excluded from Figure 9. In (A, B, C) the groove width was calculated from atoms C3’ and in (D, E) from atoms C5’. In each panel, the groove widths of all the structures are reported as dark gray crosses, and only for the indicated topology, the corresponding crosses are connected with red lines. Black lines correspond to the eight structures excluded from Figure 9 of the main text: 5J05 (A), 6JCD (B), 2MFU, 7OA3, 7OAV, 7OAW, and 7OAX (C), only the 7OA* structures (D), and 1JJP (E). The PDB structures 7OA* consist of two or four chains, corresponding to separate monomers. When the C3’ atoms are used, 7OA* (C) have the signature of hybrid3, whereas when the C5’ atoms are used, 7OA* have the signature of a parallel topology (D). In (E) 1JJP, which is a 2-tetrad interlaced dimer, with an unknown topology, has the groove-width signature, calculated from C5’ atoms, of hybrid1 topology, as well as its gc pattern (the insert). For the color code of the insert see Figure 10 in the main text.

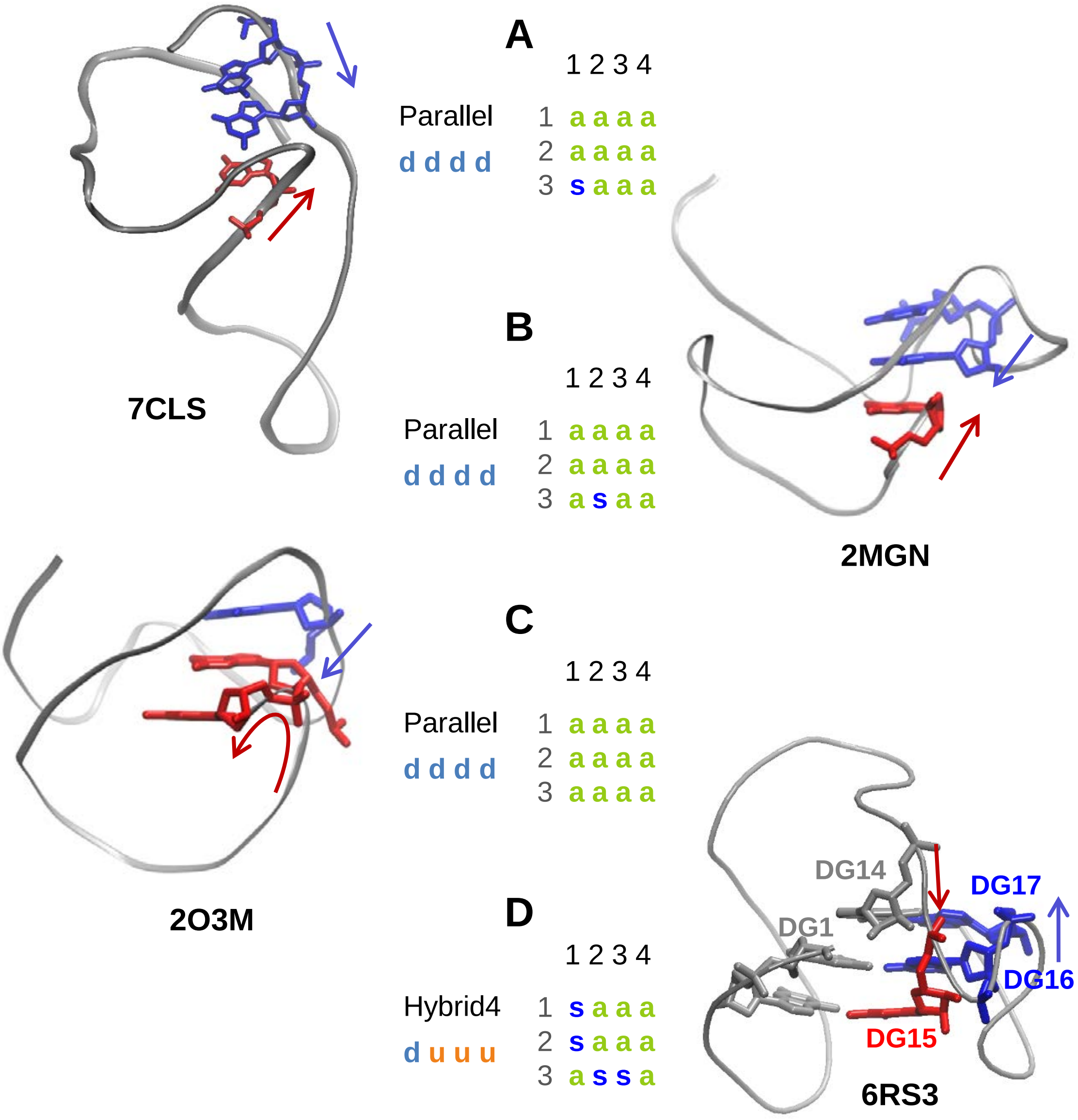

Figure S3. Example of structures with a discontinuity in a strand. The structures are presented as gray ribbons, where only the strands of interest are drawn as sticks. The regular guanosines are colored in blue and the direction of the strand is indicated by the blue arrow, whereas the guanosines that are affected by the discontinuity are colored in red and their direction is indicated by the red arrow. Near each structure, its corresponding gc pattern is shown with the same color code as in Figure 10 of the main text. (A) 7CLS: the discontinuity in strand 1 is due to a long bulge of 15 nts and the guanosine after the bulge gets back to the strand in the opposite direction (red arrow). This is why it is a *syn*-G. (B) and (C) 2MGN and 2O3M: the discontinuity is due to a 3'-bottom snapback, and therefore, the guanosines in red are the 3'-terminal nts. In (B) the snapback is in strand 2 and it consists of only one guanosine in the opposite direction to the strand (red arrow). In (C) the snapback is in strand 3 and it consists of two guanosines in the same direction as the strand. The red curved arrow indicates the direction of the loop that allows these two guanosines to insert in the right direction. (D) 6RS3: the discontinuity is in strand 1 (gray sticks), between DG1 and DG14, which affects the direction of DG15 (red) in strand 4. DG15 becomes *anti-G* because of the shortness of the 0-nt propeller loop that connects the two strands (red arrow). Strand 4, consisting of DG15 (red), DG16 (blue), and DG17 (blue), is up (blue arrow).

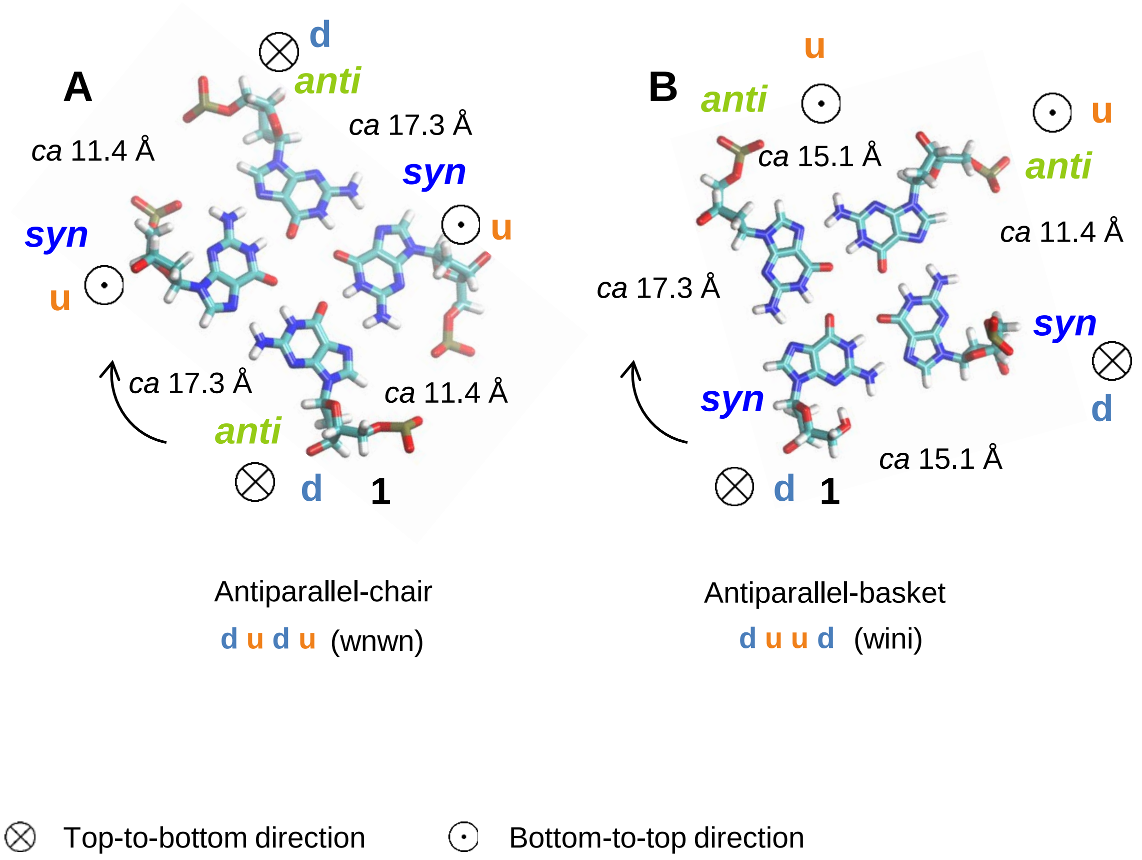

Figure S4. Determination of the topology of a regular G4 from only one tetrad. Top view of two tetrads, one starting with an *anti*-G (A), and one with a *syn*-G (B). In (A) and (B), the big curved arrow indicates the orientation of the strand numbering. Only strand 1 is indicated in bold. The topology of the G4 is determined from only one tetrad that does not contain any strand discontinuity. The first strand is down (d). From the gc progression we deduce the directions of the other strands. Then, based on the strand numbering in the clockwise orientation (the big curved arrows) we order the strand directions, and deduce the topology of the G4 from Table 1 in the main text. From this topology, we deduce the groove-width signature, and therefore, the approximate values of the average groove widths (the numbers added in each groove correspond to the groove widths calculated from atoms C5'). The atoms' color code is the following: C (cyan), N (blue), O (red), P (tan), and H (white).

In practice, following the curved arrows: In (A), the gc progression is *anti-syn-anti-syn*, therefore the corresponding strand directions are dudu, meaning that the topology is antiparallel-chair and the groove width signature is wnwn. In (B) the gc progression is *syn-anti-anti-syn*, therefore the corresponding strand directions are duud, meaning that the topology is antiparallel-basket and the groove width signature is wini. For (A) and (B), the values of the groove widths are then deduced from Table S3.

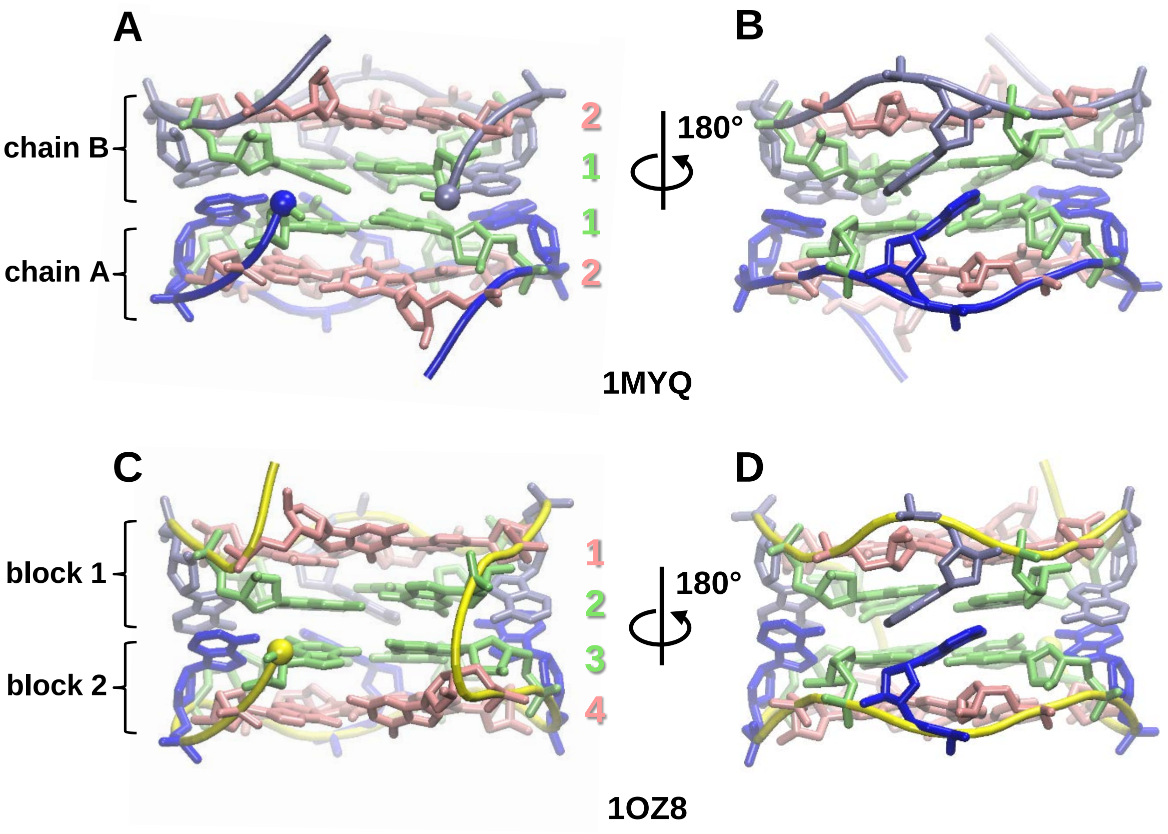

Figure S5. Comparison of the dimer 1MYQ and the two-block G4 1OZ8. (A,B) 1MYQ is shown in two opposite profile orientations. The backbone (tube) of chain A and the propeller loops of 1 nt each (in sticks) are colored in blue and those of chain B in gray. In both chains, tetrad 1 is in light green and tetrad 2 in pink. The numbers to the right in (A) are those of the tetrads. (C,D) 1OZ8 in two opposite profile orientations. The tetrads and loops are colored as in (A,B) although in 1OZ8 there is one chain made of four tetrads. The numbers of the tetrads are to the right in (C). The backbone (tube) is yellow. In (A) and (C) the C5' atom of nt 1 is represented as a small sphere. The comparison of these two panels shows the bond in 1OZ8 between the 3' extremity of chain A and the 5' extremity of chain B of 1MYQ. (B) and (D) show the opposite loop progression in the 1MYQ dimer or the two halves of 1OZ8.

Type 1: a or s<br>Only a s<br>similar a s<br>gcs<br>↓<br>Parallel	Type 2: a or s or a or s<br>Similar a s s a<br>and s a s a<br>alternate<br>gcs<br>↓<br>Hybrid	Type 3: s or a<br>Only a s<br>alternate s a<br>gcs<br>↓<br>Antiparallel
**Exceptions**		
9% of antiparallel structures<br>No CD spectra	20% of parallel structures<br>CD spectra of hybrid G4s<br><br>Parallel 1 s s s s<br>(6ERL, 6JWD, 6JWE) 2 a a a a<br>d d d d 3 a a a a	
24% of hybrid structures<br>CD spectra of parallel G4s<br><br>Hybrid1 1 a a s a<br>(5MBR) 2 a a s a<br>d d u d 3 a a s a<br><br>Hybrid2 1 a a a s<br>(5MCR, 5OV2, 6FFR, 7O1H) 2 a a a s<br>d d d u 3 a a a s<br><br>Hybrid4 1 s a a a<br>(6L8M, 6R9K, 6TCG) 2 s a a a<br>d u u u 3 s a a a	9% of antiparallel structures<br>CD spectra either of hybrid G4s or without the trough at 260 nm<br><br>Basket2 1 s s a a<br>(6F4Z, 6YEP) 2 a a s s<br>d d u u 3 a a s s<br><br>Basket2 1 s s a a<br>(2MBJ) 2 s s a a<br>d d u u 3 a a s s<br><br>Chair 1 s a s a<br>(5YEY, 6JKN) 2 a s a s<br>d u d u 3 a s a s	

Figure S6. Classification of the stacking types of regular structures, the exceptions presenting mismatches between the topologies and the stacking types, and the incidence of these mismatches on the CD spectra. In the upper three panels are exposed the three stacking types, and the major topologies where they are found. Below these panels, are exposed the exceptions, i.e., the structures where a mismatch is observed between the topology and the stacking type, with the proportions of these structures in our set. For each of these minority topologies, only the detailed gc pattern of structures with published CD spectra with mismatch is given. The format of these patterns is similar to Figure 10 in the main text. In parentheses are the PDB IDs of the concerned structures.

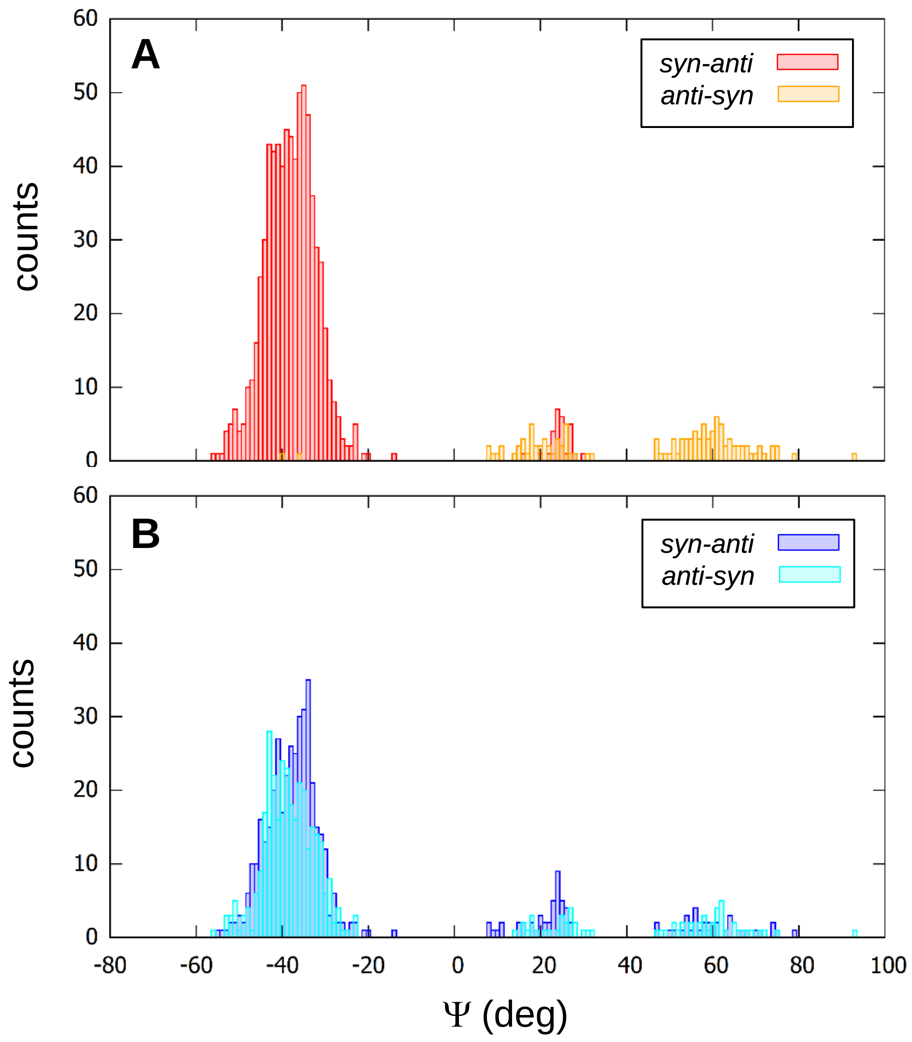

Figure S7. Comparison of the distributions of the *syn-anti* / *anti-syn* succession types. (A) The *syn-anti* distribution (red) and *anti-syn* (orange) when the reading follows the nucleotide chain direction. (B) The *syn-anti* distribution (blue) and *anti-syn* (cyan) when the reading follows the 5'→3' tetrad direction. We observe that in (A) there is a clear separation between the two populations, which is not the case in (B). The population between 0° and 40° corresponds to strands with discontinuities. The bin size is 1°.

Table S4. Characteristics of the two-block structures: topology, strand directions, handedness, and center of the range of the corresponding Ψ angles. In the strand directions column, d stands for down and u for up. The horizontal lines on the right of this column represent the tetrads, the blue arrows, the down strands and the orange arrows, the up strands. The big dot on a tetrad represents the first stem-guanine. The absence of arrows between two tetrads and the slanted-written handedness indicate the separation between the two blocks. G4-RNAs are in bold. The first six rows of the Table correspond to 4-tetrad structures and the last four rows, to 3-tetrad structures. Particularities: 2N3M, 6JCE and 6QJO have the same topology with the same strand directions, but different handednesses due to the position of the first stem-guanine in block 1 (2N3M) and block 2 (6JCE, 6QJO). 5V3F…6V9D and 7MKT have the same topology with the same strand directions, but different handednesses due to tetrads 1-2 in 7MKT, which are all *syn*-G.

Structure	Topology	Strand directions	Handedness	Centered on Ψ (deg)
2N3M	Parallel<br>/<br>Parallel	dddd<br><br>dddd 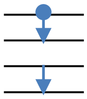	Right<br>*Right*<br>Right	20<br>20<br>20
6JCE, 6QJO	Parallel<br>/<br>Parallel	dddd<br><br>dddd 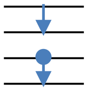	Left<br>*Left*<br>Right	-20<br>-60<br>20
2MS9, 4U5M<br>6GZ6, 7D5D<br>7D5E, 7D5F<br>7DFY	Parallel<br>/<br>Parallel	dddd<br><br>uuuu 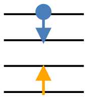	Left<br>*Left*<br>Left	-20<br>-60<br>-20
1OZ8	Parallel<br>/<br>Parallel	uuuu<br><br>dddd 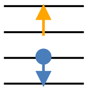	Right<br>*Left*<br>Right	20<br>-60<br>20
**6K84**	Parallel<br>/<br>Parallel	uuuu<br><br>dddd 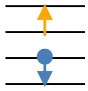	Right<br>*Right*<br>Right	20<br>-45<br>20
6KVB	Parallel<br>/<br>Hybrid2	uuuu<br><br>dddu 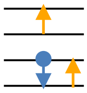	Right<br>*Right*<br>Right	20<br>-20<br>20
**2LA5, 5DE5**<br>**5DE8, 5DEA**	−/<br>Parallel	−<br>dddd 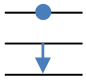	*Left*<br>Right	20<br>20
**8EYU, 8EYV**<br>**8EYW, 8F0N**	−/<br>Parallel	−<br>uuuu 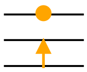	*Right*<br>Right	20<br>20
**5V3F, 6C63**<br>**6C64, 6C65**<br>**6V9B, 6V9D**	Parallel/<br>−	dddd<br>− 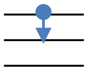	Right<br>*Right*	20<br>60
**7MKT**	Parallel/<br>−	dddd<br>− 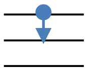	Left<br>*Right*	60<br>20

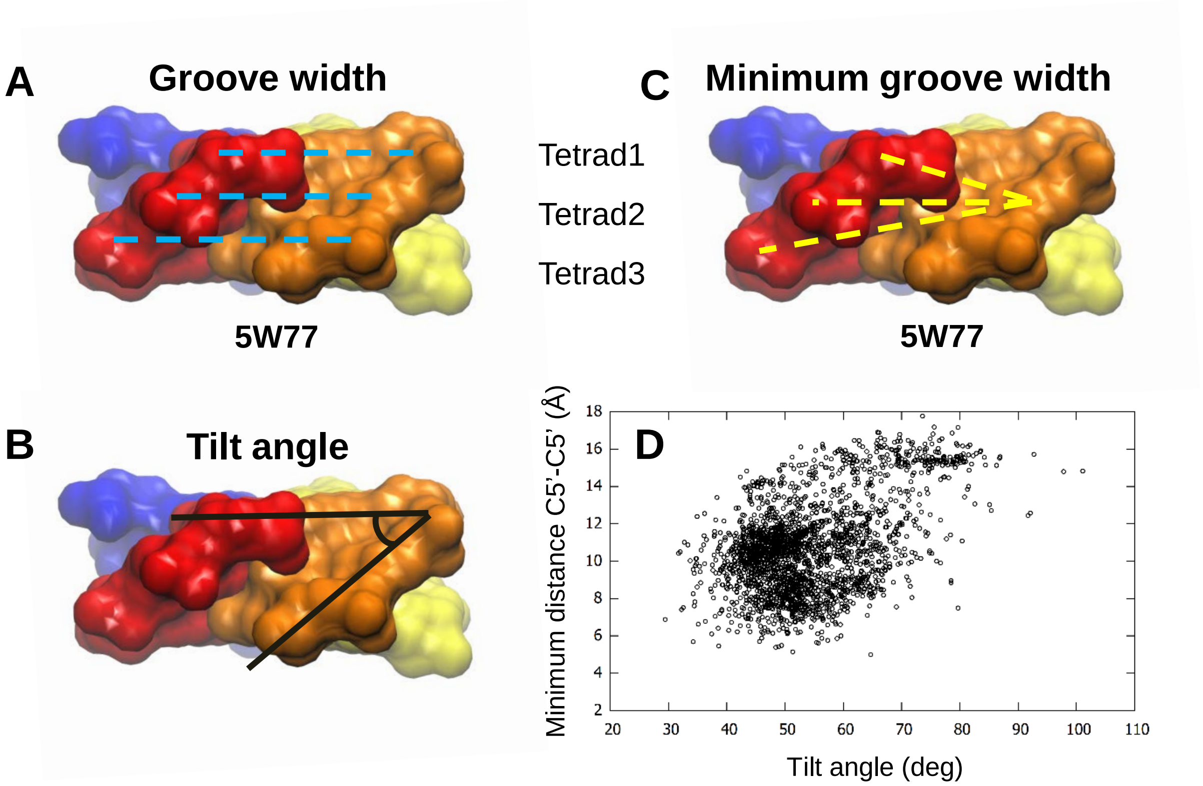

Figure S8. Minimum distances and tilt angles in regular G4s. (A-C) Representation of the accessible surface of the stem of 5W77, a 3-tetrad parallel G4. The loops are omitted for clarity. Each strand is colored differently: strand 1, in blue, strand 2 in red, strand 3 in orange, and strand 4 in yellow. (A) The groove width is calculated from the distances between atoms of the facing guanosines in each tetrad. The distances are represented as dashed blue lines. (B) The tilt angle (in black) is the angle between tetrad *i* and the part of the strand that goes from tetrad *i* to tetrad *i*+1. (C) Calculation of the minimum distance for only one guanosine at the intersection of tetrad 2 and strand 3. The three distances, which are represented as yellow dashed lines, are calculated and only the minimum value is retained. A similar calculation is performed for all guanosines in the stem. (D) The minimum C5'-C5' distances are reported *vs* the tilt angles.

Considering the example given in (B) and (C), the tilt angle at strand 3 between tetrads 1 and 2 is expected to impact the minimum distance of C5' at the intersection of strand 3 and tetrad 2. Therefore, the point corresponding to these values is reported in (D). All other points are calculated similarly, meaning that the tilt angle is between tetrads *i* and *i*+1 and the minimum C5'-C5' distance is that of tetrad *i*+1. The correlation coefficient between these values is 0.46 (p-value $<10^{-15}$).

A

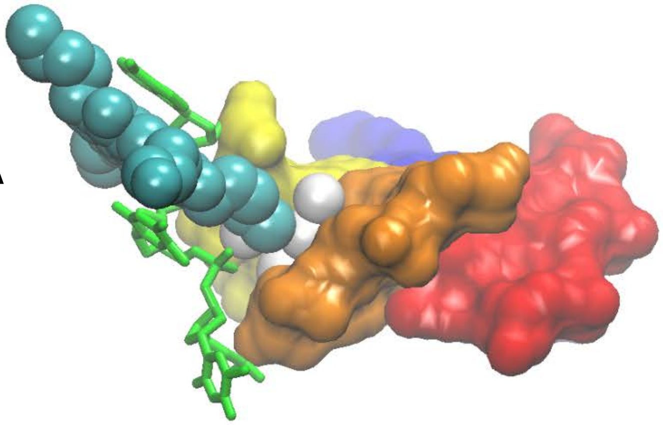

3CDM

B

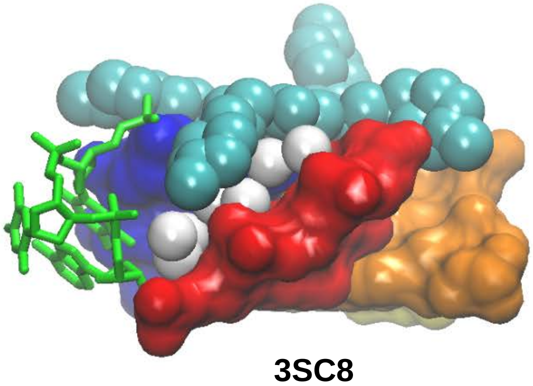

3SC8

Figure S9. Two parallel structures with a part of the ligand that seems to bind in the groove. (A) 3CDM, chain B, (B) 3SC8. The two structures are liganded to variants of naphtalene diimide. A part of the ligand seems to be bound in groove 3 in (A) and groove 1 in (B). The stem is shown as accessible surface, and colored according to the strand number: blue, red, orange, and yellow, for strands 1, 2, 3, and 4, respectively. The propeller loop in the concerned groove is drawn as green sticks, the ligand and water molecules are cyan and white hard spheres, respectively. These two examples were chosen because in Collie et al (NAR, 2015) they seemed to have, the most obviously, the ligand binding, at least partly, in the groove. However, the presence of water molecules between the ligand and the groove contradicts this.
